# The circular RNA circDLG1 promotes gastric cancer progression and anti-PD-1 resistance through the regulation of CXCL12 by sponging miR-141-3p

**DOI:** 10.1186/s12943-021-01475-8

**Published:** 2021-12-15

**Authors:** Dong-Liang Chen, Hui Sheng, Dong-Sheng Zhang, Ying Jin, Bai-Tian Zhao, Nuo Chen, Kang Song, Rui-Hua Xu

**Affiliations:** 1 State Key Laboratory of Oncology in South China, Collaborative Innovation Center for Cancer Medicine, Department of Medical Oncology, Sun Yat-sen University Cancer Center, No. 651 Dong Feng East Road, Guangzhou, 510060 PR China; 2 Research Unit of Precision Diagnosis and Treatment for Gastrointestinal Cancer, Chinese Academy of Medical Sciences, Guangzhou, 510060 P. R. China

**Keywords:** circDLG1, Gastric cancer, Proliferation, Invasion, Immune evasion

## Abstract

**Background:**

Dysregulation of circular RNAs (circRNAs) plays an important role in the development of gastric cancer; thus, revealing the biological and molecular mechanisms of abnormally expressed circRNAs is critical for identifying novel therapeutic targets in gastric cancer.

**Methods:**

A circRNA microarray was performed to identify differentially expressed circRNAs between primary and distant metastatic tissues and between gastric cancer tissues sensitive or resistant to anti-programmed cell death 1 (PD-1) therapy. The expression of circRNA discs large homolog 1 (DLG1) was determined in a larger cohort of primary and distant metastatic gastric cancer tissues. The role of circDLG1 in gastric cancer progression was evaluated both in vivo and in vitro, and the effect of circDLG1 on the antitumor activity of anti-PD-1 was evaluated in vivo. The interaction between circDLG1 and miR-141-3p was assessed by RNA immunoprecipitation and luciferase assays.

**Results:**

circDLG1 was significantly upregulated in distant metastatic lesions and gastric cancer tissues resistant to anti-PD-1 therapy and was associated with an aggressive tumor phenotype and adverse prognosis in gastric cancer patients treated with anti-PD-1 therapy. Ectopic circDLG1 expression promoted the proliferation, migration, invasion, and immune evasion of gastric cancer cells. Mechanistically, circDLG1 interacted with miR-141-3p and acted as a miRNA sponge to increase the expression of CXCL12, which promoted gastric cancer progression and resistance to anti-PD-1-based therapy.

**Conclusions:**

Overall, our findings demonstrate how circDLG1 promotes gastric cancer cell proliferation, migration, invasion and immune evasion and provide a new perspective on the role of circRNAs during gastric cancer progression.

**Supplementary Information:**

The online version contains supplementary material available at 10.1186/s12943-021-01475-8.

## Background

Gastric cancer is one of the most commonly diagnosed cancers and a cause of cancer-related death worldwide [1]. Although great progress has been made in the treatment of gastric cancer in recent years, the prognosis is still poor, especially for advanced-stage patients. Tumor progression and distant metastasis are the main causes of death. For metastatic gastric cancer patients, chemotherapy is still the main therapeutic strategy. In recent years, increasing evidence has shown that immune evasion is essential for tumor survival and development [2, 3]. It has been reported that in the tumor microenvironment, tumor cells can recruit immunosuppressive cells, such as CD4^+^ T cells, to disrupt the cytotoxic functions of CD8^+^ T cells [4, 5]. In addition, programmed death ligand 1 (PD-L1), a B7 family ligand, can bind to its receptor programmed death-1 (PD-1) to influence tumor-specific T cells, induce apoptosis and inhibit the activity of CD8^+^ T cells, leading to immune evasion in tumors. Clinically, an increasing number of studies have shown that blocking the PD-1 checkpoint with an anti-PD-1 antibody is an efficient immunotherapy approach in different cancers [6, 7]. Our group confirmed the clinical benefit of PD-1 antibody therapy in gastric cancer patients [8]. However, most gastric cancer patients are resistant to anti-PD-1 therapy, and only some patients benefit from this treatment [9]. PD-L1 has been proven to be a predictive biomarker of anti-PD-1 efficacy in several tumor types. However, the predictive role of PD-L1 expression in gastric cancer is controversial [10]. The KENOTE-061 and KENOTE-062 trials showed better survival in patients with PD-L1-positive tumors after pembrolizumab treatment [11, 12]. On the other hand, data from the Checkmate032, JAVELIN Gastric 300, and ATTRCTION-2 trials did not support the concept of PD-L1 positivity as a predictive biomarker of anti-PD-1 efficacy [13–15]. Therefore, a better understanding of the molecular mechanisms that contribute to gastric cancer progression and immune evasion is critical for developing effective therapeutic strategies for this disease.

Circular RNAs (circRNAs) are a class of recently identified noncoding RNAs or protein-coding RNAs that are characterized by covalently closed loops without 5′ to 3′ polarity or polyadenylation (polyA) tails [16]. Previous reports have found that circRNAs are generally derived from the back-splicing of pre-mRNA transcripts and are mainly located in the cytoplasm [17, 18]. Studies have demonstrated that circRNAs are conserved, stable and abundant in different tumor cells and tissues [19]. Increasing studies have indicated that circRNAs participate in multiple physiological and pathological processes by modulating gene expression, apoptosis, the cell cycle, cell migration and invasion [20]. Our previous study identified a circRNA signature that can predict postoperative recurrence in stage II-III colon cancer [21]. Mechanistically, circRNAs can exert their functions in different ways, such as sponging miRNAs, interacting with RNA-binding proteins, and translating proteins [22–24]. For instance, our previous study found that circUBXN7 expression was downregulated in bladder cancer, and forced circUBXN7 expression could suppress cell growth and invasion by sponging miR-1247 to enhance B4GALT3 expression in bladder cancer cells [25]. Rong et al. [26] demonstrated that circPSMC3 could inhibit the proliferation and metastasis of gastric cancer. Han et al. [27] reported that circMTO1 modulated the progression of hepatocellular carcinoma through the regulation of p21 expression by sponging miR-9. Hsiao et al. [28] verified that the circRNA CCDC66 promoted the progression and metastasis of colon cancer. Although the molecular mechanisms of dysregulated circRNA-associated pathways have been extensively explored, the role of circRNAs in gastric cancer progression and immune evasion remains poorly understood.

In this study, to identify circRNAs involved in gastric cancer progression and immune evasion, we performed a circRNA array using primary and distant metastatic tumor tissues as well as tissues sensitive or resistant to anti-PD-1 therapy. We found that the circRNA circDLG1 (hsa_circ_0008583), which is derived from the DLG1 gene, was significantly upregulated in distant metastatic tissues and primary gastric cancer tissues resistant to anti-PD-1 therapy. More importantly, circDLG1 expression was significantly associated with an aggressive tumor phenotype and adverse prognosis in gastric cancer patients who received anti-PD-1 therapy. In addition, ectopic expression of circDLG1 promoted in vitro cell proliferation, invasion, immune evasion, and in vivo tumorigenesis and metastasis in immunocompetent mice. Mechanistically, circDLG1 could directly interact with miR-141-3p, acting as a miRNA sponge to increase the expression of the miR-141-3p target gene chemokine 12 (CXCL12), thereby promoting the progression of gastric cancer. Thus, circDLG1 might be a promising therapeutic target and biomarker for gastric cancer.

## Methods

### Human tissue samples and cell lines

Fresh-frozen or paraffin-embedded tissues were obtained from advanced-stage gastric cancer patients who underwent gastroscopy and biopsy, fine needle biopsy, or palliative surgery and received anti-PD-1 therapy at Sun Yat-sen University Cancer Center from August 2018 to October 2019. These patients were all included in a real-world study of PD-1 antibody therapy in gastric cancer (No: NCT04086888). A total of 126 gastric cancer patients were enrolled. Eighty-two of these patients had primary tumor tissues available. Seventy-three patients could be evaluated for the efficacy of PD-1 antibody therapy, among which 30 patients had tissues available (including primary tissues, adjacent normal tissues, and distant metastatic tissues). These patients were evaluated every 3-4 cycles of therapy and followed up regularly. The response rate was evaluated based on RECIST 1.1 guidelines. The tumor response included complete response (CR), partial response (PR), stable disease (SD), and progressive disease (PD). The objective response rate (ORR) was defined as the percentage of patients who achieved CR and PR. The clinical and pathological parameters, including age, sex, tumor size, tumor cell differentiation, peritoneal metastasis, and Lauren’s classification, were collected from patient records. This study was approved by the ethics committee of the Sun Yat-sen University Cancer Center, and informed consent was obtained from all patients. Overall survival (OS) was calculated from the start of PD-1 antibody treatment to the date of death or last contact, and progression-free survival (PFS) was calculated from the start of PD-1 antibody treatment to the date of progression or death.

Human gastric cancer cell lines (HGC27, BGC823, MKN45, MKN28, SGC7901, AGS), the normal gastric epithelial cell line GES-1, the murine gastric cancer cell line MFC and human embryonic kidney (HEK) 293 T cells were purchased from the Shanghai Institute of Cell Biology, Chinese Academy of Science (Shanghai, China). All cell lines were cultured according to the provider’s instructions.

### CircRNA microarray assays

To identify the potential circRNAs involved in gastric cancer progression and anti-PD-1 resistance, 3 patients who responded to anti-PD-1 (experienced PR) therapy with PFS of more than 10 months and 6 patients who were nonresponsive to anti-PD-1 (2 SD and 4 PD) therapy with PFS of less than 5 months were selected for circRNA microarray analysis.

The circRNA microarray was performed by Kangchen Biotech (Shanghai, China). All sample preparation and microarray hybridization steps were conducted according to the instructions of Arraystar (Rockville, MD, USA). In brief, circRNAs were enriched by removing the linear RNAs with RNase R treatment, and the enriched circRNAs were then amplified and transcribed into fluorescent cRNA with a random priming method (Arraystar Super RNA Labeling Kit). Then, the labeled cRNAs were hybridized onto Human circRNA Array V2 (8 × 15 k, Arraystar). Finally, the arrays were scanned using the Agilent Scanner G2505C and analyzed with Agilent Feature Extraction software (version 11.0.1.1). The random variance model was used to identify the differentially expressed genes. The paired t-test was used to calculate the *P* value. The threshold for differentially expressed genes was fold change ≥2.0 and a P value ≤0.05.

### Quantitative real-time polymerase chain reaction (qRT–PCR)

Total RNA was isolated from human gastric cancer tissues and cell lines using TRIzol reagent (Sigma–Aldrich, St. Louis, MO, USA) according the manufacturer’s instructions. Reverse transcription of mRNA and miRNA was performed using random primers and stem–loop primers, respectively. qRT–PCR was conducted using a TaqMan Universal Master Mix II kit on a Bio–Rad CFX96 qPCR system, and fold changes were determined by using the relative quantification 2^-ΔΔCT^ method. The nuclear and cytoplasmic fractions of cells were separated using the PARIS Kit (Life Technologies) according to the manufacturer’s instructions. RNA was extracted from both fractions. Then, qRT–PCR was performed to determine the expression ratios of specific RNA molecules between the nuclear and cytoplasmic fractions. GAPDH and U6 served as cytoplasmic and nuclear markers, respectively. The primers used are presented in Additional file 1: Table S1.

### Western blotting analysis

Western blotting analysis was performed according to a previously described method [29]. Briefly, proteins were extracted from gastric cancer cell lines, and the protein concentration was calculated using the Pierce BCA protein assay kit (Thermo Scientific, Rockford, IL, USA). The following antibodies were used in this study: anti-GAPDH (CST, #2118 L) and anti-CXCL12 (CST, #3740S).

### Immunohistochemistry (IHC) analysis

The IHC analysis was performed according to the method we described previously [30]. Briefly, the paraffin-embedded tissue blocks were cut into 4-μm slides. Immunostaining images were captured using a microscope (Leica, Germany). The immunoreactivity in each tissue section was assessed by two pathologists, and the degree of positivity was evaluated according to the percentage of positive tumor cells. The following antibodies were used: anti-CXCL12 (CST, 97958S; 1:200 dilution), anti-PD-L1 (22C3 pharmDX; Dako, Carpinteria, CA, USA), and anti-CD33 (clone PWS44; 1:200 dilution; Leica Biosystems, Nussloch, Germany). PD-L1 positivity was defined as a membrane staining intensity ≥1% in the tumor cells or tumor infiltrating immune cells. The median value of PD-L1 expression was used as the cutoff value to stratify high and low PD-L1 expression.

### Flow cytometry

The detailed flow cytometry procedure was performed as previously described [31]. The following antibodies were used: anti-mouse IFNγ (BD Biosciences), anti-mouse CD45 (BD Biosciences), anti-mouse Gr-1 (TONBO Biosciences), anti-mouse Ly6G (TONBO Biosciences), anti-mouse ly6C (BD Biosciences), anti-mouse CD11b (TONBO Biosciences), anti-mouse F4/80 (BioLegend), anti-human CD11b (TONBO Biosciences), anti-human CD33 (BD Biosciences), and anti-human HLA-DR (BD Biosciences). Detailed information on the antibodies is provided in Additional file 2: Table S2. Briefly, the cells were digested and suspended as single cells, washed with PBS, and then resuspended in cell stabilizing buffer (BioLegend cat. No. 420201); the supernatant was discarded, and the sample was centrifuged at 350 g for 5 min. Then, 5 μl of Human TruStain FcX™ (Fc Receptor Blocking Solution, BioLegend Cat. No. 422301) and the antibodies were added and cultured for 15-20 min at room temperature. Finally, the cells were assessed by flow cytometry. For intracellular staining of IFNγ, fixation buffer (BioLegend Cat. No. 420801) was added and incubated for 20 min at room temperature. Then, the cells were washed with Intracellular Staining Perm Wash Buffer (BioLegend Cat. No. 421002), resuspended and centrifuged. IFNγ antibody was added, and the cells were cultured for 20 min and finally assessed by flow cytometry.

### Fluorescence in situ hybridization (FISH) assays

The FISH assay was performed according to previously described methods [25]. Briefly, cells were hybridized with Cy3-labeled circDLG1 probe and Cy5-labeled miR-141-3p probe (GenePharma, China) for 12 h at 37 °C. Then, the nuclei were counterstained using 4′,6-diamidino-2-phenylindole (DAPI) (Yeasen, Shanghai, China). Finally, the cells were viewed and captured under a ZEISS LSM800 confocal microscope (Carl Zeiss AG, Germany).

### Cell proliferation, colony formation, migration and invasion assays

Cell counting kit-8 (CCK-8) and colony formation assays were performed to assess the cell proliferation ability. Transwell assays were conducted to evaluate cell migration and invasion abilities. The details of these assays were described in our previous study [32].

### RNA immunoprecipitation (RIP) and luciferase activity assays

The RIP assay was conducted using the Magna RIP RNA-bing Protein Immunoprecipitation kit (Millipore, USA) according to the provider’s protocol. In brief, cell lysates were cultured with Dynabeads-coated IgG antibody (Millipore, USA) or AGO2 antibody (Cell Signaling Technology, USA) for 12 h at 4 °C. The purified RNA was subjected to qRT–PCR to detect the enriched circDLG1 and miRNA.

For the luciferase activity assay, potential binding sites were predicted using StarBase v3.0 and TargetScanHuman 7.2. Gastric cells were cotransfected with pGL-luc-circDLG1, pGL-luc-CXCL12 3′-UTR, and miR-141-3p mimics or negative control mimics for 48 h, and luciferase activity was detected using the dual-luciferase reporter assay system (Promega, USA) according to the manufacturer’s instructions.

### circDLG1 knockdown or overexpression transfection experiment

Small hairpin RNAs (shRNAs) targeting the junction region of the circDLG1 sequence and the circDLG1-overexpressing lentiviral vector were synthesized by Geneseed Biotech Co., Ltd. (Guangzhou, China). Gastric cancer cell lines were transfected with circDLG1 shRNA or the circDLG1-overexpressing lentiviral vector following the manufacturer’s instructions according to a previously described method [25].

### In vivo tumorigenesis and metastasis assays

The in vivo tumorigenesis and metastasis experiments in BALB/c nude mice and C57BL/6 mice were approved by the Animal Experiment Ethics Committee of Sun Yat-sen University Cancer Center. The mice were purchased from the Shanghai Institute of Material Medicine (Shanghai, China) and were fed according to the provider’s instructions. The experiments were conducted as described in our previous study [33]. Briefly, to evaluate the in vivo tumorigenesis effect of circDLG1, MFC-sh-circDLG1 and MFC-sh-NC cells (1 × 10^6^ cells/mouse) were inoculated subcutaneously into the flanks of two groups of BALB/c nude mice and C57BL/6 mice (ten for each cell group). Tumor size was measured every 4 days, and tumor volume was estimated. After 4 weeks, the mice were sacrificed, and the tumors were excised. To investigate the effect of circDLG1 on tumor metastasis, MFC-sh-circDLG1 and MFC-sh-NC cells (2 × 10^6^ cells/mouse) were inoculated into the tail vein of two groups of BALB/c nude mice and C57BL/6 mice (ten for each cell group). Four weeks later, the mice were sacrificed, and the lungs were excised and paraffin embedded. Consecutive sections (4 μm) were made and stained with hematoxylin-eosin. The micrometastases in the lungs were examined and counted under a dissecting microscope.

### Mouse xenograft anti-PD-1 therapy experiment

To evaluate the effect of CXCL12 on immune evasion and the efficacy of anti-PD-1 therapy in vivo, an in vivo xenograft experiment was conducted in C57BL/6 mice. In brief, four groups of mice were implanted subcutaneously in the left flanks with MFC-sh-NC, and two groups of mice were implanted subcutaneously in the left flanks with MFC-sh-CXCL12 cells (ten mice for each group, 1 × 10^6^ cells/mouse). The MFC-sh-NC implanted mice were treated with 0.9% normal saline (NS), anti-PD-1 antibody, AMD3100 (a CXCR4 inhibitor), and the combination of AMD3100 and anti-PD-1 antibody. MFC-sh-CXCL12-implanted mice were treated with 0.9% normal saline (NS) and an anti-PD-1 antibody. A mouse anti-PD-1 antibody was purchased from Bio X Cell (West Lebanon, NH, USA). AMD3100 was purchased from Selleck (Shanghai, China) and dissolved in phosphate-buffered saline (PBS). The mice were intraperitoneally injected with 0.9% NS, AMD3100, or anti-PD-1 antibody according to a previously described method [34]. The mice were sacrificed at 4 weeks or before becoming moribund. The survival time was defined as the date of first therapy to the date of sacrifice.

### RNA in situ hybridization (ISH), sphere formation assay, and RNA sequencing (RNA-seq) analysis

The methods for ISH, sphere formation assay and RNA-seq are described in Additional file 3: Supplementary materials and methods.

### Statistical analysis

All data are presented as the mean ± standard deviation unless otherwise noted. Statistical analyses were performed with SPSS 17.0 software (SPSS Inc., Chicago, IL, USA) or GraphPad Prism 7 (GraphPad Software, Inc., La Jolla, CA, USA). For comparisons between groups, Student’s t-test, chi-squared test, one-way ANOVA, and Pearson correlation analysis were used, as appropriate. PFS was analyzed using the Kaplan–Meier method with the log-rank test, and Cox’s proportional hazard regression model was performed to evaluate the independent prognostic indicators. A *P*-value of < 0.05 was considered significant.

## Results

### Identification of gastric cancer progression- and immune evasion-associated circRNAs through microarray

**Fig. 1:**
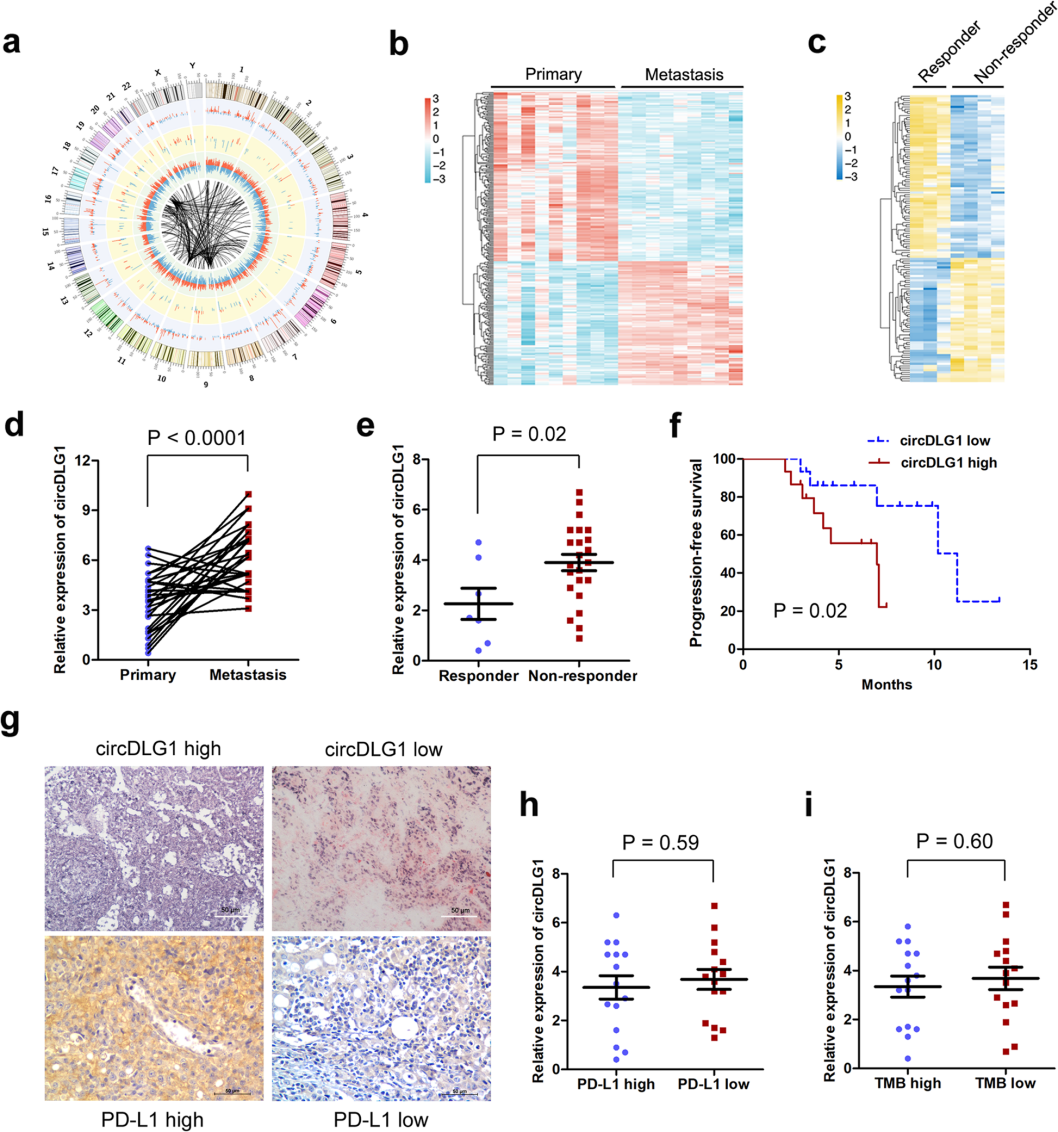
Identification of gastric cancer progression- and immune evasion-associated circRNAs through microarray. **a** A circRNA microarray was performed to identify differentially expressed circRNAs in gastric cancer tissues. The circus plot shows the distribution and expression profiles of the detected and differentially expressed circRNAs located on human chromosomes, as well as potential miRNAs interacting with differentially expressed circRNAs (inner, mRNA; median, miRNAs; outer, circRNAs). **b** The heatmap presents the significantly expressed circRNAs in primary and paired distant metastatic gastric cancer tissues (*n* = 9). **c** The heatmap presents the significantly expressed circRNAs in primary gastric cancer tissues responsive (*n* = 3) or resistant (*n* = 4) to anti-PD-1 therapy. **d** Relative expression of circDLG1 in primary and distant metastatic gastric cancer tissues measured by qRT–PCR (*n* = 30) (*P* < 0.001). The relative expression of circDLG1 was determined using the relative quantification 2^-ΔΔ^CT method. Student’s t-test. The data are representative of three technical replicates. **e** Relative expression of circDLG1 in primary gastric cancer tissues responsive (*n* = 7) and resistant (*n* = 23) to anti-PD-1 therapy (*P* = 0.002). The relative expression of circDLG1 was measured by qRT–PCR and calculated using the relative quantification 2^-ΔΔ^CT method. Paired Student’s t-test. The data are representative of three technical replicates. **f** The 30 patients were divided into circDLG1 expression-high (*n* = 15) and circDLG1 expression-low (n = 15) groups based on the median expression level of circDLG1. Kaplan–Meier analysis was performed to evaluate progression-free survival in the two groups. Patients with high circDLG1 expression presented significantly worse survival than those with low circDLG1 expression (*P* = 0.02). **g** ISH analysis of circDLG1 expression (blue staining, positive expression; red staining, negative) and IHC analysis of PD-L1 expression in gastric cancer tissues. Scale bar, 50 μm. **h** circDLG1 expression in the PD-L1 high and PD-L1 low groups measured by qRT–PCR (*P* = 0.59). The relative expression of circDLG1 was determined using the relative quantification 2^-ΔΔ^CT method. Student’s t-test. **i** The expression of circDLG1 in the TMB high and TMB low groups measured by qRT–PCR (*P* = 0.60). The relative circDLG1 expression was determined using the relative quantification 2^-ΔΔ^CT method. Student’s t-test

To identify the circRNA expression profiles involved in gastric cancer progression and immune evasion, we performed a circRNA microarray analysis on 9 paired gastric cancer tissues (primary tissues and paired distant metastatic lesions) from patients who received anti-PD-1 treatment. The circus plot shows the distribution and expression profiles of the detected and differentially expressed circRNAs on human chromosomes, as well as potential miRNAs interacting with differentially expressed circRNAs (Fig. 1a). The heatmap presents the significantly expressed circRNAs in gastric cancer and paired distant metastatic tissues (Fig. 1b). To identify the potential circRNAs involved in anti-PD-1 resistance, we compared circRNA profiling in the primary tissues of 3 patients who were responsive (experienced PR) and 4 patients who were nonresponsive (experienced PD) to anti-PD-1 therapy (Fig. 1c). To select a circRNA that plays a critical role in gastric cancer progression and immune evasion, circRNAs (hsa_circ_0008583, has_circ_0002387) that were upregulated in both distant metastatic lesions and tissues resistant to anti-PD-1 therapy were identified. However, only hsa_circ_0008583 (derived from exons 13, 14, 15 and 16 of DLG1, thus designated circDLG1) was confirmed to be upregulated in distant metastatic lesions and primary gastric cancer tissues resistant to anti-PD-1 therapy in a larger cohort of patients (Fig. 1d and e). Moreover, circDLG1 expression was higher in primary tumor tissues than in adjacent normal tissues, although the difference was not significant (Additional file 4: Fig. S1). Kaplan–Meier analysis showed that circDLG1 expression was significantly associated with PFS in gastric cancer patients treated with anti-PD-1 therapy, and patients with low circDLG1 expression presented with significantly better prognosis than those with high circDLG1 expression (Fig. 1f). The patients were divided into two groups (high expression and low expression) based on the mean circDLG1 expression level from qRT–PCR. circDLG1 expression was significantly associated with tumor size and peritoneal metastasis. However, no association was observed between circDLG1 expression and age, sex, tumor cell differentiation, or Lauren’s classification (Additional file 5: Table S3). Several previous reports indicated that PD-L1 expression and tumor mutation burden (TMB) were associated with the anti-PD-1 response in gastric cancer [8, 9]. We questioned whether circDLG1 expression is associated with PD-L1 expression and TMB. ISH analysis was also used to confirm circDLG1 expression in gastric cancer tissues (blue staining indicates positive expression; red staining indicates negative), and IHC was performed to detect PD-L1 expression in gastric cancer tissues (Fig. 1g). However, no significant correlation was found between circDLG1 expression and PD-L1 expression or TMB (Fig. 1h and i).

Taken together, these results demonstrate that circDLG1 might play an important role in gastric cancer progression and immune evasion.

### Characterization of circDLG1 in gastric cancer

**Fig. 2:**
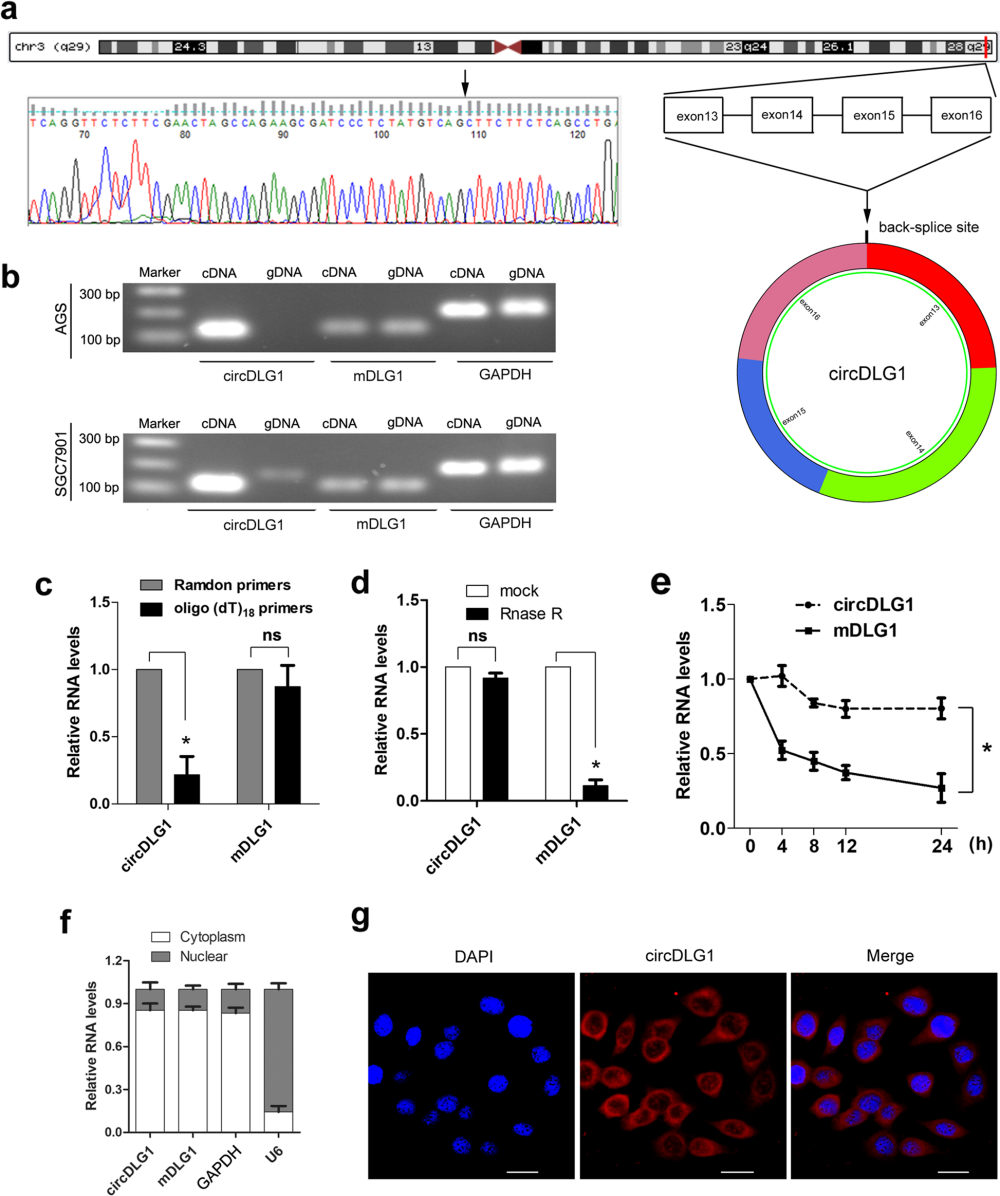
Characterization of circDLG1 in gastric cancer. **a** A scheme illustrating the production of circDLG1. **b** CircDLG1 was validated by RT–PCR, and its back splicing junction was verified by Sanger sequencing. The existence of circDLG1 was validated in AGS and SGC7901 cell lines by RT–PCR. GAPDH was used as a linear control. **c** Random hexamer or oligo (dT)18 primers were used in the reverse transcription experiments. The relative RNA levels were analyzed by qRT–PCR and normalized to the value obtained using random hexamer primers. *P < 0.05, Student’s t-test; the experiment was repeated three times. **d** The relative RNA levels were analyzed by qRT–PCR and normalized to the value detected in the mock group (AGS cells without transfection of RNase R). *P < 0.05, Student’s t-test; the experiment was repeated three times. **e** The relative RNA levels of circDLG1 and mDLG1 were analyzed by qRT–PCR after treatment with actinomycin D at the indicated time points in SGC7901 cells. *P < 0.05, two-way ANOVA; the experiment was repeated three times. **f** The expression of circDLG1 and mDLG1 was measured by qRT–PCR in the nuclear and cytoplasmic fractions, respectively. GAPDH and U6 were used as positive controls for the cytoplasm and nucleus, respectively. **g** RNA FISH for circDLG1 in the gastric cancer cell line SGC7901. Nuclei were stained with DAPI. Scale bar, 20 μm. The data are representative of three independent replicates

According to the circBase database and UCSC, circDLG1 (circRNA ID: hsa_circ_0008583) is derived from exons 13, 14, 15 and 16 of DLG1, which is located on chromosome 3 and has a length of 496 nucleotides (Fig. 2a). CircDLG1 was amplified by using divergent primers and confirmed by Sanger sequencing in AGS and SGC7901 gastric cancer cells (Fig. 2b), and PCR analysis of reverse-transcribed RNA (cDNA) and genomic DNA (gDNA) was performed. The results showed that the divergent primers could amplify products from cDNA but not from gDNA (Fig. 2b). To further confirm the characteristics of circDLG1, we used random hexamer or oligo (dT)_18_ primers for reverse transcription of RNA from gastric cancer cells. The relative expression of circDLG1 was significantly decreased, while the expression of DLG1 mRNA was not decreased when oligo (dT)_18_ was used (Fig. 2c). This finding indicates that circDLG1 has no poly A tail. Moreover, circDLG1 was resistant to RNase R (a highly processive 3′ to 5′ exoribonuclease that degrades linear RNAs but not circRNAs), while DLG1 mRNA was sensitive to RNase R (Fig. 2d). In addition, SGC7901 cells were treated with actinomycin D (an inhibitor of transcription), and RNA was extracted at the indicated times for the detection of circDLG1. The results demonstrated that the half-life of circDLG1 was significantly longer than that of DLG1 mRNA (Fig. 2e). Furthermore, the qRT–PCR and FISH results indicated that circDLG1 is mainly located in the cytoplasm (Fig. 2f and g). These results demonstrate that circDLG1 is a stable and abundant circRNA transcript.

### CircDLG1 promotes gastric cancer progression

**Fig. 3:**
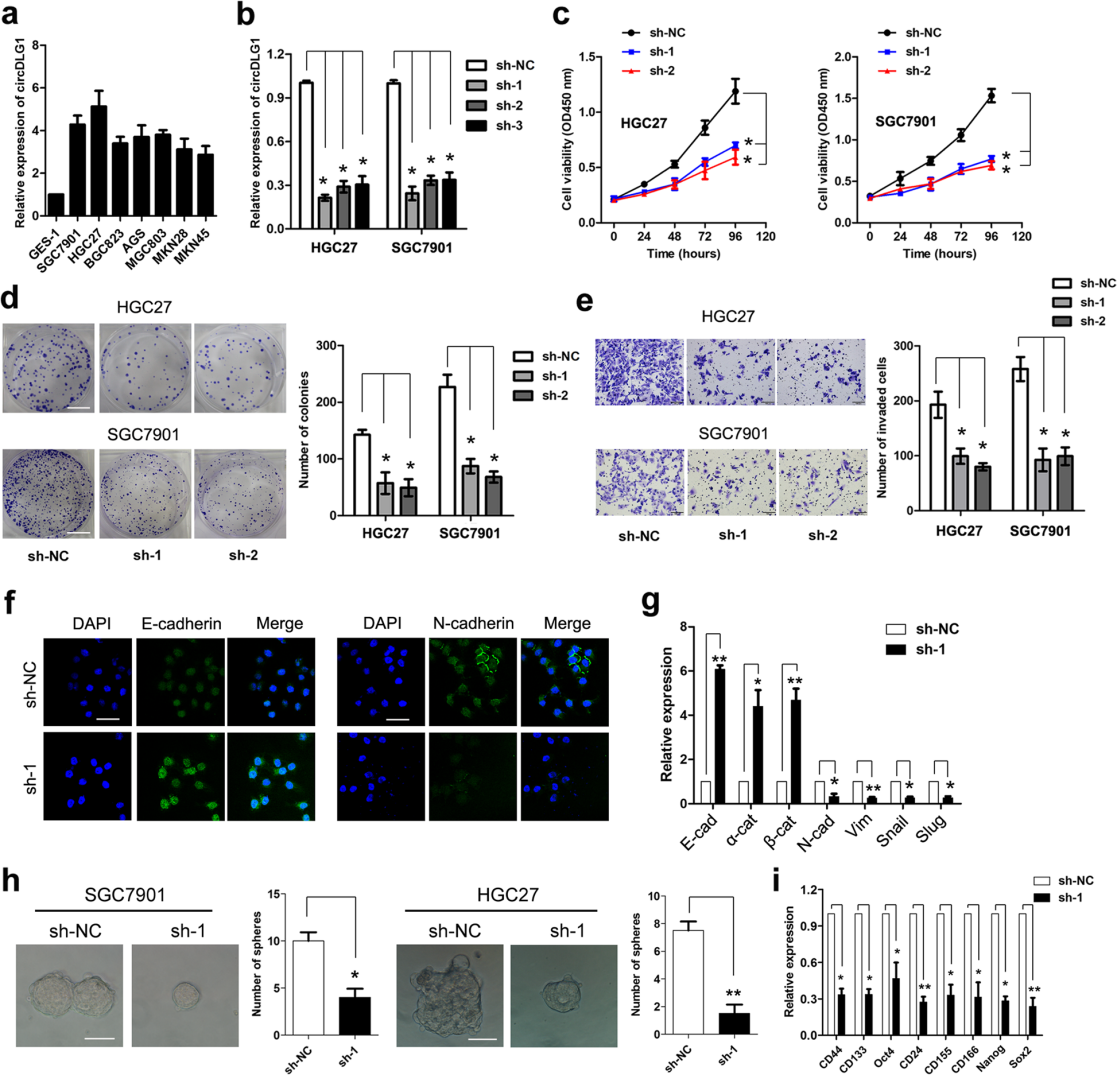
CircDLG1 promotes gastric cancer progression. **a** The relative expression of circDLG1 in gastric cancer cell lines and the gastric normal epithelial cell line GES-1 measured by qRT–PCR. **b** The expression of circDLG1 in HGC27 and SGC7901 cells treated with shRNAs (sh-circDLG1#NC, sh-circDLG1#1, sh-circDLG1#2, sh-circDLG1#3) measured by qRT–PCR. *P < 0.05, one-way ANOVA test for the comparison of the three groups, followed by Student’s t-test for the comparison of sh-1 and sh-1 group with sh-NC group, respectively; multigroup comparisons were adjusted using the Bonferroni method. The experiment was repeated three times. **c** CCK-8 assay of the gastric cancer cell lines HGC27 and SGC7901 treated with shRNAs. *P < 0.05, two-way ANOVA test, multigroup comparisons were adjusted using the Bonferroni method. The experiment was repeated three times. **d** Colony formation assays of the gastric cancer cell lines HGC27 and SGC7901 treated with shRNAs. *P < 0.05, one-way ANOVA test for the comparison of the three groups, followed by Student’s t-test for the comparison of sh-1 and sh-1 group with sh-NC group, respectively; multigroup comparisons were adjusted using the Bonferroni method. The experiment was repeated three times. Scale bar, 50 mm. **e** Transwell assay of the gastric cancer cell lines HGC27 and SGC7901 treated with shRNAs. *P < 0.05, one-way ANOVA test for the comparison of the three groups, followed by Student’s t-test for the comparison of sh-1 and sh-2 group with sh-NC group, respectively; multigroup comparisons were adjusted using the Bonferroni method. The experiment was repeated three times. Scale bar, 100 μm. **f** Immunofluorescence analysis of E-cadherin and N-cadherin expression after knockdown of circDLG1 in SGC7901 cells. Scale bar, 50 μm. **g** qRT–PCR analysis of epithelial and mesenchymal markers after knockdown of circDLG1 in SGC7901 cells. *P < 0.05, **P < 0.01, Student’s t-test; the experiment was repeated three times. **h** Sphere formation assay in SGC7901 and HGC27 cells after knockdown of circDLG1. *P < 0.05, **P < 0.01, Student’s t-test; the experiment was repeated three times. Scale bar, 100 μm. **i** qRT–PCR analysis of stemness-associated markers after knockdown of circDLG1 in SGC7901 cells. *P < 0.05, **P < 0.01, Student’s t-test; the experiment was repeated three times

To explore the biological function of circDLG1 in gastric cancer, we first measured circDLG1 expression in gastric cancer cell lines and the gastric normal epithelial cell line GES-1. The results showed that circDLG1 expression was upregulated in gastric cancer cell lines compared with GES-1 gastric normal epithelial cells (Fig. 3a). We designed three shRNA plasmids to target the unique back-splice junction. Back-splice junction-specific shRNAs (sh-1, sh-2, sh-3) effectively knocked down circDLG1 expression in HGC27 and SGC7901 cells (Fig. 3b). In vitro CCK-8 and colony formation assays indicated that knockdown of circDLG1 significantly inhibited the proliferation and colony formation ability of HGC27 and SGC7901 cells (Fig. 3c and d). In addition, transwell assays indicated that knockdown of circDLG1 significantly inhibited the invasion ability of HGC27 and SGC7901 cells (Fig. 3e). Moreover, considering that epithelial-mesenchymal transition (EMT) plays an important role in the process of cancer progression and invasion, we explored the effects of circDLG1 on the EMT and stemness of gastric cancer cells. Immunofluorescence analysis showed that knockdown of circDLG1 significantly increased the expression of E-cadherin while decreasing the expression of N-cadherin (Fig. 3f). qRT–PCR analysis indicated that knockdown of circDLG1 markedly increased the expression of epithelial markers (E-cadherin, α-catenin, β-catenin) while decreasing the expression of mesenchymal markers (N-cadherin, vimentin, Snail, Slug) (Fig. 3g). IHC showed that the expression of E-cadherin decreased, whereas vimentin expression increased in tissues with high expression of circDLG1 (Additional file 6: Fig. S2). In addition, sphere formation assays showed that knockdown of circDLG1 markedly inhibited sphere formation (Fig. 3h), and qRT–PCR analysis indicated that knockdown of circDLG1 significantly inhibited the expression of stemness-associated markers (CD44, CD133, Oct4, CD24, CD155, CD166, Nanog, Sox2) (Fig. 3i).

### CircDLG1 promotes tumor progression via increased infiltration of myeloid-derived suppressor cells (MDSCs)

**Fig. 4:**
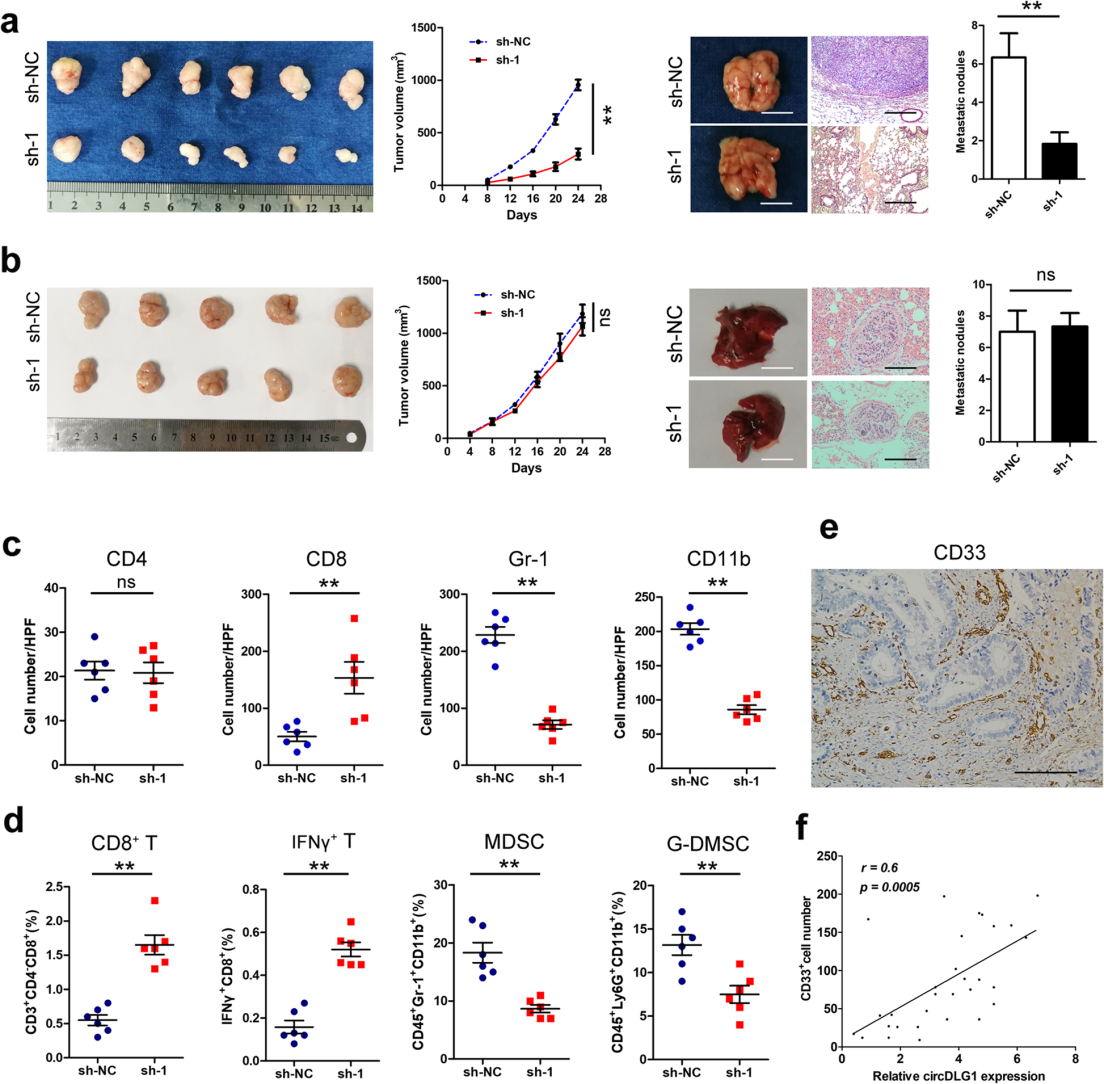
CircDLG1 promotes tumor progression by increasing the infiltration of MDSCs. **a** C57BL/6 mice were injected with MFC-sh-NC or MFC-sh-circDLG1 cells, and the tumor volume and tumor metastasis are shown. The mice were sacrificed at 4 weeks after injection. Left: the tumor size was measured, and the tumor volume was calculated every 4 days (*n* = 10 mice per group), **P < 0.05, two-way ANOVA. Right: the lungs of the mice were removed and paraffin embedded, consecutive sections (4 μm) were made and stained with hematoxylin-eosin, and the micrometastases in the lungs were examined and counted under a dissecting microscope (*n* = 10 mice per group), **P < 0.05, Student’s t-test. All experiments were repeated three times. Scale bar, left: 5 mm; right: 100 μm. **b** BALB/c nude mice were injected with MFC-sh-NC or MFC-sh-circDLG1 cells. The mice were sacrificed at 4 weeks after injection. Left: the tumor size was measured, and the tumor volume was calculated every 4 days (*n* = 10 mice per group). ns, no significance, two-way ANOVA. Right: The lungs of the mice were removed and paraffin embedded. Consecutive sections (4 μm) were made and stained with hematoxylin-eosin, and the micrometastases in the lungs were examined and counted under a dissecting microscope (*n* = 10 mice per group). ns, no significance, Student’s t-test. All experiments were repeated three times. Scale bar, left: 5 mm; right: 100 μm. **c** IHC analysis was performed using subcutaneous tumors for CD4, CD8, Gr-1, and CD11b (Gr-1 and CD11b are mouse MDSC markers). HPF, high-power visual field. ns, no significance; **P < 0.05. Student’s t-test. **d** Flow cytometric analysis of immune cells in subcutaneous tumors. **P < 0.05, Student’s t-test; the experiment was repeated three times. **e** The expression of CD33 (a marker of human MDSCs) was detected by IHC in human gastric cancer tissues. Scale bar, 50 μm. **f** The correlation between circDLG1 expression measured by qRT–PCR and CD33^+^ cells. *n* = 30, *P < 0.05, Pearson correlation analysis

We generated MFC-sh-circDLG1 cell lines through the stable transduction of circDLG1-targeting shRNA vectors into the MFC murine gastric cancer cell line. qRT–PCR analysis confirmed that knockdown of circDLG1 significantly decreased circDLG1 expression in MFC cells (Additional file 7: Fig. S3). To elucidate the function of circDLG1 in vivo, MFC-sh-circDLG1 cells were injected into immunocompetent mice subcutaneously or via the tail vein. The results showed that knockdown of circDLG1 significantly inhibited tumor progression and metastasis compared with the control treatment (Fig. 4a). In contrast, no significant difference was observed in tumor progression or metastasis between the MFC-sh-circDLG1 and MFC-control groups of immunodeficient mice (Fig. 4b). These results demonstrated that circDLG1 promotes gastric cancer progression and metastasis and might be related to tumor immune evasion. IHC analysis showed that MFC-sh-circDLG1-derived tumors were associated with increased CD8^+^ tumor-infiltrating lymphocytes (TILs) and decreased infiltration of Gr-1- and CD11b-positive MDSCs (Gr-1 and CD11b are mouse MDSC markers) (Fig. 4c, Additional file 8: Fig. S4). Moreover, flow cytometric analysis of immune cells in subcutaneous tumors showed that knocking down circDLG1 significantly increased the number of CD8^+^ T cells and IFNγ^+^ cells but significantly decreased the number of MDSCs and granulocytic (G)-MDSCs (Fig. 4d). We further evaluated the relationship between circDLG1 and MDSCs in gastric cancer tissues. The expression of CD33 (a marker of human MDSCs) was detected by IHC, and the results showed a significant correlation between circDLG1 and CD33^+^ cells (Fig. 4e and f). These results demonstrated that circDLG1 promotes gastric cancer progression by inducing MDSC infiltration.

### CircDLG1 upregulates CXCL12 to promote cell progression and immune evasion in gastric cancer

To further explore the underlying molecular mechanism by which circDLG1 promotes gastric cancer progression and immune evasion, gene expression profiling was performed in gastric cancer cells with or without knockdown of circDLG1. SGC7901 cells were transfected with circDLG1 shRNA, and the expression levels of mRNAs were detected by RNA sequencing in these cells (Additional file 9: Fig. S5a). Gene annotation enrichment analysis showed that differentially expressed genes involved in T cell migration, EMT and immune responses were significantly enriched based on Gene Ontology (GO) analysis, and TNF signaling and the PI3K-Akt signaling pathway were significantly enriched based on Kyoto Encyclopedia of Genes and Genomes (KEGG) analysis (Additional file 9: Fig. S5b). The circRNA/mRNA microarray dataset was analyzed, and the correlation between circDLG1 and mRNAs was determined. The results showed that CXCL12 had the highest correlation coefficient. In addition, CXCL12 was one of the most significantly decreased genes by knockdown of circDLG1 in gastric cancer cells based on the RNA sequencing assay. Thus, we speculated that there might be an association between circDLG1 and CXCL12. qRT–PCR analysis showed that knockdown of circDLG1 significantly decreased the expression of CXCL12, whereas ectopic expression of circDLG1 significantly increased CXCL12 expression in gastric cancer cell lines (Additional file 9: Fig. S5c and d). Western blot analysis confirmed that knockdown of circDLG1 significantly decreased the protein level of CXCL12 in SGC7901 and HGC27 gastric cancer cells (Additional file 9: Fig. S5e). Moreover, the decreased level of CXCL12 caused by CXCL12 knockdown could be restored by circDLG1 ectopic expression (Additional file 9: Fig. S5f). Accordingly, the suppressed cell migration ability caused by CXCL12 knockdown could be restored by circDLG1 ectopic expression in gastric cancer cells (Additional file 9: Fig. S5g). Furthermore, circDLG1 expression was significantly associated with CXCL12 in gastric cancer tissues (Additional file 9: Fig. S5h). These data indicated that circDLG1 exerts its effect at least partly by regulating CXCL12 expression.

### CircDLG1 functions as a sponge for miR-141-3p to regulate CXCL12 expression

**Fig. 5:**
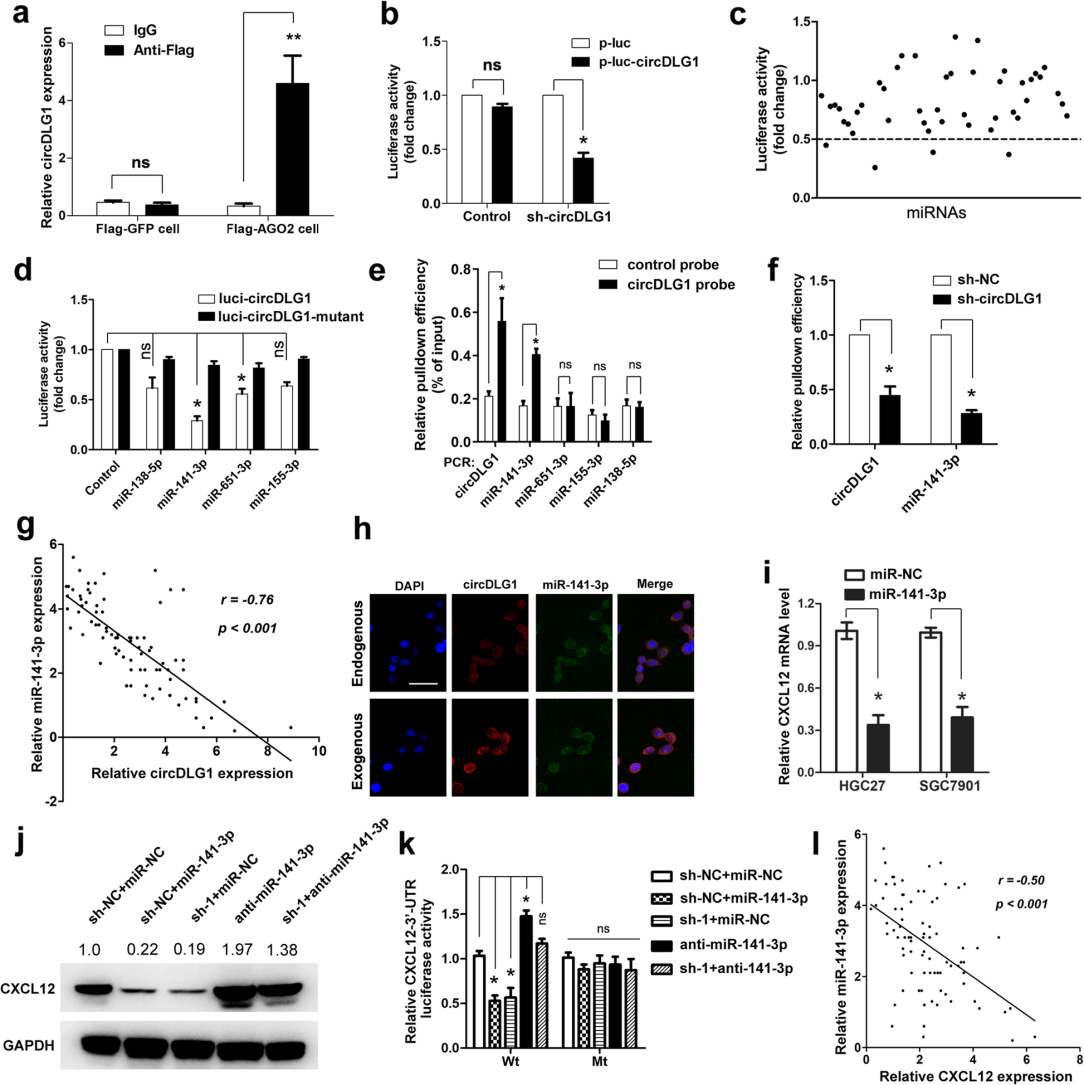
CircDLG1 functions as a sponge for miR-141-3p to regulate CXCL12 expression. **a** The results of RNA immunoprecipitation for AGO2 in SGC7901 cells stably expressing Flag-AGO2 and Flag-GFP. **P < 0.01, Student’s t-test; the experiment was repeated three times. **b** The luciferase activity of the circDLG1 reporter after transfection of circDLG1 shRNA in SGC7901 cells. *P < 0.05, Student’s t-test; the experiment was repeated three times. **c** A luciferase screen with a miRNA mimic library was constructed in SGC7901 cells. **d** The luciferase activity of the circDLG1 reporter after transfection with four miRNAs (miR-141-3p, miR-138-5p, miR-651-3p, and miR-155-3p) in SGC7901 cells. *P < 0.05, one-way ANOVA test for the comparison of all groups, followed by Student’s t-test for the comparison of miR-138-5p, miR-141-3p, miR-651-3p, and miR-155-3p with the control group. Multigroup comparisons were adjusted using the Bonferroni method. The experiment was repeated three times. **e** RIP analysis with a biotin-labeled circDLG1 probe in SGC7901 cells. *P < 0.05, Student’s t-test; the experiment was repeated three times. **f** RIP analysis with a biotin-labeled circDLG1 probe in SGC7901 cells treated with sh-circDLG1. *P < 0.05, Student’s t-test; the experiment was repeated three times. **g** The association between the expression of circDLG1 and miR-141-3p in gastric cancer tissues. *n* = 82, *P* < 0.001, Pearson correlation analysis. **h** RNA FISH assay of circDLG1 and miR-141-3p in gastric cancer cells. Scale bar, 50 μm. **i** The mRNA level of CXCL12 in gastric cancer cells after the expression of miR-141-3p. *P < 0.05, Student’s t-test; the experiment was repeated three times. **j** SGC7901 cells and SGC7901 cells with circDLG1 knockdown (SGC7901-sh-circDLG1#1/SGC7901-sh-NC) were treated with miR-NC, miR-141-3p, or miR-141-3p inhibitor, the protein was extracted, and western blot analysis of CXCL12 protein levels was performed. **k** SGC7901 cells and SGC7901 cells with circDLG1 knockdown (SGC7901-sh-circDLG1#1/SGC7901-sh-NC) were cotransfected with miR-NC, miR-141-3p, or miR-141-3p inhibitor and pGL-luc-CXCL12 3′-UTR (wild-type) or pGL-luc-CXCL12 3′-UTR (mutant-type). Luciferase activity was detected using the dual-luciferase reporter assay system (Promega, USA) according to the manufacturer’s instructions. *P < 0.05, one-way ANOVA test for the comparison of all groups, followed by Student’s t-test for the comparison of the other groups with the sh-NC + miR-NC group. Multigroup comparisons were adjusted using the Bonferroni method. The experiment was repeated three times. **l** The association between miR-141-3p expression and CXCL12 expression in gastric cancer tissues. *n* = 82, *P* < 0.001, Pearson correlation analysis

Previous studies have reported that circRNAs can act as miRNA sponges to regulate the expression of protein-coding genes. In the present study, we found that circDLG1 was mainly located in the cytoplasm, which indicated that it might function as a miRNA sponge. Interestingly, by using the online bioinformatics tools miRanda and TargetScan, 46 miRNAs were predicted to be potential targets of circDLG1 (Additional file 10: Table S4). We first explored the occupancy of AGO2 in the region of circDLG1. The results of RNA immunoprecipitation for AGO2 in SGC7901 cells stably expressing Flag-AGO2 and Flag-GFP showed that endogenous circDLG1 pulled down by Flag-AGO2 was significantly enriched, suggesting that circDLG1 is incorporated into the RNA-induced silencing complex (RISC) (Fig. 5a). To identify the miRNAs that can interact with circDLG1, a circDLG1 fragment was amplified and inserted downstream of a luciferase reporter gene. Transfection of circDLG1 shRNA significantly decreased the luciferase activity of the circDLG1 reporter (Fig. 5b). To further identify the key miRNAs that can bind to circDLG1, a luciferase screen with a miRNA mimic library was performed. A total of 46 miRNA mimics were cotransfected with the circDLG1 luciferase reporter into SGC7901 cells (Fig. 5c). The luciferase activity of the circDLG1 reporter was significantly reduced by four miRNAs (miR-141-3p, miR-138-5p, miR-651-3p, miR-155-3p) (Fig. 5d). In addition, RNA precipitation (RIP) analysis with a biotin-labeled circDLG1 probe was conducted to evaluate the key miRNAs that can interact with circDLG1. Compared with the negative control, miR-141-3p, but not the other three miRNAs, was enriched by the biotin-labeled circDLG1 probe (Fig. 5e), and the enrichment of miR-141-3p by circDLG1 was reduced when circDLG1 was knocked down in gastric cancer cells (Fig. 5f). In addition, an inverse association was observed between the expression of circDLG1 and miR-141-3p in gastric cancer tissues (Fig. 5g). Furthermore, the RNA FISH assay demonstrated that miR-141-3p colocalized with both endogenous and exogenous circDLG1 in gastric cancer cells (Fig. 5h). These data demonstrated that circDLG1 could function as a miR-141-3p sponge in gastric cancer cells.

To evaluate whether circDLG1 could regulate CXCL12 expression by sponging miR-141-3p, we first confirmed the regulation of CXCL12 by miR-141-3p. The results showed that ectopic expression of miR-141-3p could significantly decrease the mRNA level of CXCL12 in gastric cancer cells (Fig. 5i). Moreover, Western blot analysis showed that ectopic expression of miR-141-3p or knockdown of circDLG1 markedly reduced the protein level of CXCL12, inhibition of miR-141-3p significantly increased the protein level of CXCL12, and the increased level of CXCL12 by miR-141-3p inhibition could be partly restored by circDLG1 knockdown (Fig. 5j). Accordingly, the luciferase activity assay showed that ectopic expression of miR-141-3p or knockdown of circDLG1 significantly reduced the luciferase activity of wild-type CXCL12-3′-UTR, inhibition of miR-141-3p significantly increased the luciferase activity of wild-type CXCL12-3′-UTR, and the increased luciferase activity of wild-type CXCL12-3′-UTR by miR-141-3p inhibition could be partly restored by circDLG1 knockdown; however, no significant change in luciferase activity was observed when the CXCL12-3′-UTR was mutated (Fig. 5k). In addition, miR-141-3p expression was inversely associated with CXCL12 expression in gastric cancer tissues (Fig. 5l). These results indicated that circDLG1 could function as a miR-141-3p sponge to regulate CXCL12 expression.

### CXCL12 is associated with tumor progression and anti-PD-1 resistance in gastric cancer

It has been reported that CXCL12 plays an important role in cancer progression and immune evasion [35, 36], and we questioned whether CXCL12 is implicated in gastric cancer progression and anti-PD-1 therapy resistance. The potential role of CXCL12 was further explored in gastric cancer cells. In vitro experiments showed that knockdown of CXCL12 significantly inhibited the invasion ability of SGC7901 and HGC27 cells, whereas ectopic expression of CXCL12 significantly increased the invasion ability of AGS cells (Additional file 11: Fig. S6a). Moreover, knockdown of CXCL12 markedly decreased the expression of N-cadherin but increased the expression of E-cadherin (Additional file 11: Fig. S6b).

**Fig. 6:**
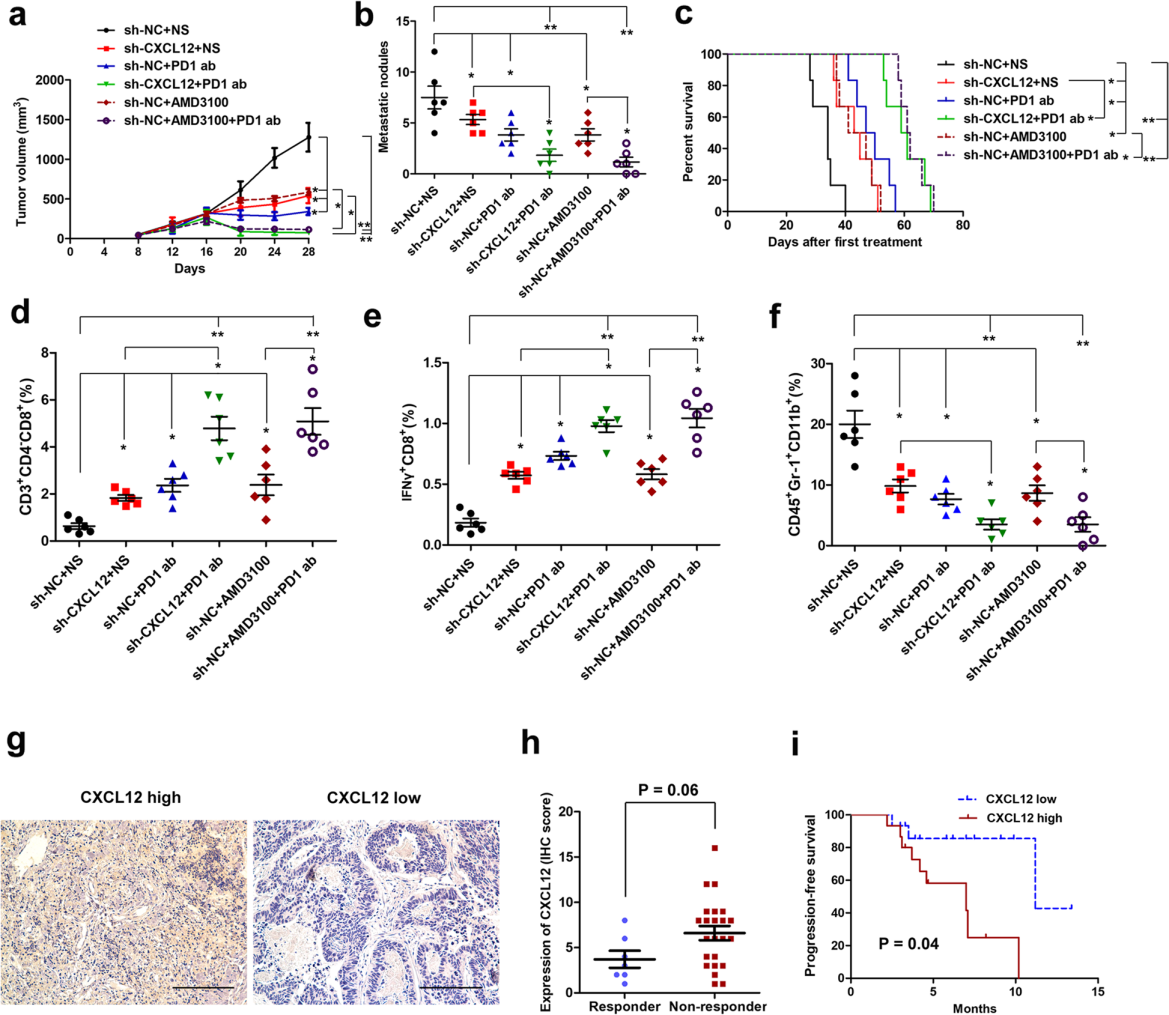
CXCL12 is associated with anti-PD-1 resistance in gastric cancer. **a** C57BL/6 mice were injected with different cells and treated with 0.9% normal saline (NS), AMD3100 (CXCR4 inhibitor), or anti-PD-1, and the tumor volume was measured at the indicated times. *P < 0.05, **P < 0.01, two-way ANOVA test, multigroup comparisons were adjusted using the Bonferroni method. **b** C57BL/6 mice were injected with different cells and treated with 0.9% NS, AMD3100, or anti-PD-1. The mice were sacrificed, and metastatic nodules in the lung were determined. *P < 0.05, **P < 0.01, one-way ANOVA test for the comparison of all groups, followed by one-way ANOVA test for the comparison of more than two groups or Student’s t-test for the comparison of two groups (marked in the figure). Multigroup comparisons were adjusted using the Bonferroni method. **c** Cumulative survival time was estimated by the Kaplan–Meier method, and the log-rank test was applied to compare the groups. Multigroup comparisons were adjusted using the Bonferroni method, *P < 0.05, **P < 0.01. **d**-**f** Flow cytometric analysis of immune cells in subcutaneous tumors was performed to evaluate the number of CD8^+^ T cells, IFNg^+^ cells, and MDSCs in different groups. *P < 0.05, **P < 0.01, one-way ANOVA test for the comparison of all groups, followed by one-way ANOVA test for the comparison of more than two groups or Student’s t-test for the comparison of two groups (marked in the figure). Multigroup comparisons were adjusted using the Bonferroni method. **g** Representative image of CXCL12 expression evaluated by IHC in gastric cancer tissues. Scale bar, 50 μm. **h** The association between CXCL12 expression and anti-PD-1 efficacy. *P* = 0.06, Student’s t-test. **i** The survival curve in mice with different CXCL12 expression levels. *P* = 0.04, Kaplan–Meier survival analysis

To evaluate the effect of CXCL12 on anti-PD-1 efficacy, MFC-sh-NC or MFC-sh-CXCL12 cells were injected into C57BL/6 mice subcutaneously or via the tail vein. Then, the mice were treated with 0.9% normal saline (NS), AMD3100 (CXCR4 inhibitor), or anti-PD-1. The results showed that compared with those in the control group, knockdown of CXCL12 significantly inhibited tumor growth and metastasis, and anti-PD-1 treatment further reduced tumor growth and metastasis in C57BL/6 mice (Fig. 6a and b). Likewise, blocking CXCL12/CXCR4 using AMD3100 significantly inhibited tumor growth and metastasis, and the combination of AMD3100 and anti-PD-1 further reduced tumor growth and metastasis (Fig. 6a and b). Moreover, Kaplan–Meier analysis indicated that knockdown of CXCL12 or AMD3100 treatment could significantly prolong the OS of mice, and anti-PD-1 therapy could further increase the OS in mice (Fig. 6c). In addition, flow cytometric analysis of immune cells in subcutaneous tumors showed that knockdown of CXCL12, AMD3100 treatment and anti-PD-1 therapy significantly increased the number of CD8^+^ T cells and IFNγ^+^ cells but decreased the number of MDSCs (Fig. 6d-f). We then evaluated the association of CXCL12 expression and the anti-PD-1 response in gastric cancer patients. The results showed that high expression of CXCL12 had a strong trend to be associated with resistance to anti-PD-1 therapy, and patients with high CXCL12 expression presented with adverse survival after anti-PD-1 therapy (Fig. 6g-i).

## Discussion

Increasing evidence has indicated that circRNAs are involved in different diseases [37, 38]. Moreover, many circRNAs are expressed in a cell type-specific or tissue-specific manner [39], indicating that they might play important biological functions. The dysregulation of circRNAs has been found in several pathological processes, including cardiac hypertrophy, neurological disorders and tumor development [38, 40]. By analyzing the circRNA expression profiles in primary and paired distant metastatic gastric cancer tissues as well as tissues that respond to or are resistant to anti-PD-1 therapy, we found that circDLG1 (circRNA ID: hsa_circ_0008583) was upregulated in distant metastatic lesions and tissues resistant to anti-PD-1 therapy, and high circDLG1 expression was associated with worse PFS in gastric cancer patients treated with anti-PD-1 therapy. This finding indicated that circDLG1 might play an important role in gastric cancer progression and anti-PD-1 resistance.

Further biological experiments demonstrated that circDLG1 could promote cell proliferation, migration, EMT, and stem cell formation. Moreover, an in vivo assay showed that circDLG1 induced gastric cancer progression and metastasis in immunocompetent mice but not in immunodeficient mice. This finding implies that circDLG1 might be involved in the regulation of immunity. Indeed, further analysis showed that knockdown of circDLG1 increased CD8^+^ T cells while decreasing MDSCs. Previous reports have indicated that EMT characteristics are associated with immune evasion and anti-PD-1 resistance in different tumors [41–43]. Our results showed that circDLG1 is critical for the two hallmark processes of tumors, EMT and immune evasion.

Previous studies have shown that several biomarkers, including MSI, PD-L1 expression, TMB, EBV status and Pold/Pole, might be associated with the response to anti-PD-1 therapy [9, 44]. Our previous study showed that the circulating tumor DNA landscape could predict the efficacy of immune checkpoint inhibitors in some gastric cancer patients [45]. However, more biomarkers are needed to identify gastric cancer patients who can benefit from anti-PD-1 therapy. In this study, we found that circDLG1 upregulated the expression of CXCL12. Indeed, previous studies have indicated that CXCL12 is involved in the progression and metastasis of gastric cancer [46]. Moreover, increasing evidence indicates that CXCL12 can induce immune evasion by recruiting infiltrating MDSCs into the tumor microenvironment [36]. In this study, we showed that knockdown of CXCL12 could inhibit the invasion ability and improve the sensitivity of gastric cancer to anti-PD-1 therapy. CXCL12 expression is associated with the PFS of patients who receive anti-PD-1 therapy. This finding is in accordance with previous studies of other tumor types [34, 47]. Taken together, these data demonstrated that CXCL12 might be used as a biomarker to predict gastric cancer resistance to anti-PD-1 therapy. Furthermore, the CXCL12/CXCR4 axis has been found to play an important role in anti-PD-1 resistance in other cancer types [34]. Blocking CXCL12/CXCR4 using AMD3100 (a CXCR4 inhibitor) could improve the efficacy of anti-PD-1 in melanoma [47]. Interestingly, we found that AMD3100 treatment could significantly improve the efficacy of anti-PD-1 therapy in gastric cancer in vivo. This finding indicates that targeting CXCL12 might be a promising strategy to improve anti-PD-1 efficacy in gastric cancer.

Increasing evidence indicates that circRNAs can act as miRNA sponges to regulate the expression of protein-coding genes. For example, the circRNA ACVR2A suppresses bladder cancer cell proliferation and metastasis through the miR-626/EYA4 axis [48]; the circRNA circ-RanGAP1 regulates VEGFA expression by targeting miR-877-3p to facilitate gastric cancer invasion and metastasis [49], and the circRNA circCCDC9 acts as a sponge for miR-6792-3p to inhibit gastric cancer progression [50]. In the present study, we found that circDLG1 was mainly located in the cytoplasm of gastric cancer cells, and RIP and RNA pulldown assays showed that circDLG1 could interact with miR-141-3p. Luciferase activity assays confirmed the direct relationship between miR-141-3p and circDLG1. In addition, RNA FISH assays indicated that miR-141-3p and circDLG1 could colocalize in the cytoplasm. These data demonstrate that circDLG1 could sponge miR-141-3p to regulate the expression of CXCL12. Our study has several limitations. First, the sample size was relatively small, and further validation is needed using a larger cohort of patients. Second, we did not detect circDLG1 in the plasma, which might be due to the degradation of RNA after a long storage time of the samples. Further validation is needed to confirm the existence of circDLG1 in plasma or exosomes. Third, the mouse model could not fully reflect the tumor immune microenvironment of the patients, and further study using a patient-derived xenograft model is needed to confirm our results.

## Conclusion

In conclusion, we identified a novel circRNA, circDLG1, that is upregulated in gastric cancer distant metastatic lesions and tissues resistant to anti-PD-1 therapy. Further functional studies showed that circDLG1 could promote gastric cancer proliferation, invasion, immune evasion and anti-PD-1 therapy resistance. Clinically, circDLG1 expression was associated with adverse prognosis in gastric cancer patients treated with anti-PD-1 therapy. Additional experiments indicated that circDLG1 acts as a miR-141-3p sponge to regulate the expression of CXCL12, which could induce MDSC infiltration to impair the function of CD8^+^ T cells. Our study revealed the significance of the circDLG1/CXCL12 axis in the immune evasion and anti-PD-1 resistance of gastric cancer.

## Supplementary Information

**Additional file 1: Table S1.** Primers used in the paper were listed.

**Additional file 2: Table S2.** The information for antibodies used in flow cytometry.

**Additional file 3.** Supplementary methods.

**Additional file 4: Figure S1.** RT–qPCR analysis of circDLG1 expression in primary gastric cancer tissues and adjacent normal tissues (*n* = 30, *P* = 0.14). *P* = 0.14, Paired Student’s t-test. The data are representative of three technical replicates.

**Additional file 5: Table S3.** The association between clinicopathologic parameters and circDLG1 expression in 30 gastric cancer patients.

**Additional file 6: Figure S2.** IHC analysis of E-cadherin and vimentin expression in primary gastric cancer tissues with high and low circDLG1 expression. Scale bar, 100 μm.

**Additional file 7: Figure S3.** circDLG1 expression in MFC cells treated with shRNAs. *P < 0.05, one-way ANOVA test for the comparison of the three groups, followed by Student’s t-test for the comparison of sh-1 and sh-2 group with sh-NC group, respectively; multigroup comparisons were adjusted using the Bonferroni method. The experiment was repeated three times.

**Additional file 8: Figure S4.** IHC analysis of CD4, CD8, Gr-1, CD11b expression in MFC-sh-circDLG1-derived tumors and MFC-sh-NC-derived tumors. Scale bar, 100 μm.

**Additional file 9: Figure S5.** CircDLG1 upregulates CXCL12 to promote cell progression and immune evasion in gastric cancer. a, A gene expression profile was performed in gastric cancer cells with or without circDLG1 knockdown. SGC7901 cells were transfected with circDLG1 shRNA, and the expression level of mRNAs was detected by RNA sequencing in SGC7901 cells. b, Gene annotation enrichment analysis showed that differentially expressed genes involved in T cell migration, EMT and immune responses were significantly enriched based on the GO analysis, and TNF signaling and the PI3K-Akt signaling pathway were significantly enriched based on the KEGG analysis. c, qRT–PCR analysis of CXCL12 expression in SGC7901 gastric cancer cells after knockdown of circDLG1. **P < 0.01, Student’s t-test; the experiment was repeated three times. d, qRT–PCR analysis of CXCL12 expression in SGC7901 gastric cancer cells after ectopic expression of circDLG1. **P < 0.01, Student’s t-test; the experiment was repeated three times. e, Western blot analysis of CXCL12 in SGC7901 and HGC27 gastric cancer cells after knockdown of circDLG1. f, Western blot analysis of CXCL12 in SGC7901 cells treated with different vectors. g, The cell migration ability of SGC7901 gastric cancer cells treated with different vectors. Scale bar, 100 μm. h, The association between circDLG1 expression and CXCL12 expression as measured by qRT–PCR in gastric cancer tissues. *n* = 30, *P* = 0.002, Pearson correlation analysis.

**Additional file 10: Table S4.** The potential miRNAs that could bind to has_circ_0008583 (circDLG1).

**Additional file 11: Figure S6.** CXCL12 is associated with an aggressive tumor phenotype in gastric cancer cells. a, The invasion ability of the gastric cancer cell lines SGC7901 and HGC27 after knockdown of CXCL12. *P < 0.05, one-way ANOVA test for the comparison of the three groups, followed by Student’s t-test for the comparison of sh-1 and sh-2 group with sh-NC group, respectively; multigroup comparisons were adjusted using the Bonferroni method. The invasion ability of AGS cells treated with the CXCL12 ectopic expression vector. *P < 0.05, Student’s t-test. Scale bar, 100 μm. b, FISH analysis of N-cadherin and E-cadherin expression after knockdown of CXCL12 in SGC7901 cells. Scale bar, 50 μm.

## Data Availability

All the data are included in this published article and additional files.

## Abbreviations

circRNA: Circular RNA
DLG1: Discs large homolog 1
FISH: Fluorescence in situ hybridization
IHC: Immunohistochemistry
MDSCs: Myeloid-derived suppressor cells
qRT–PCR: Quantitative real-time polymerase chain reaction
PD-1: Programmed death-1
PD-L1: Programmed death ligand 1
EMT: Epithelial-mesenchymal transition
RIP: RNA immunoprecipitation
PR: Partial remission
SD: Stable disease
PD: Progressive disease
ISH: In situ hybridization
CCK-8: Cell Counting Kit-8
HPF: High-power visual field
