# Supplementary material for: The circular RNA circDLG1 promotes gastric cancer progression and anti-PD-1 resistance through the regulation of CXCL12 by sponging miR-141-3p
Source: Mol Cancer. 2021 Dec 15;20:166. doi: 10.1186/s12943-021-01475-8 (PMC8672580; doi:10.1186/s12943-021-01475-8)
Supplement: Supplementary file 1 — Additional file 1: Table S1. Primers used in the paper were listed. [file 12943_2021_1475_MOESM1_ESM.docx]

**Table S1** Primers used in the paper were listed.

| **Gene** | **Primer** | **Sequence(5′-3′)** |
| --- | --- | --- |
| **Primers for qRT-PCR** | |  |
| Circ_0008583 | forward | 5’- TGTTGCACAATATCGACCTGAA -3’ |
|  | reverse | 5’- CAGGCTGAGAAGAAGCTGAC -3’ |
| N-cadherin | forward | 5’-GGTGGAGGAGAAGAAGACCAG-3’ |
|  | reverse | 5’-GGCATCAGGCTCCACAGTG-3’ |
| Vimentin | forward | 5’-GAGAACTTTGCCGTTGAAGC-3’ |
|  | reverse | 5’-GCTTCCTGTAGGTGGCAATC-3’ |
| Snail | forward | 5’-CCTCCCTGTCAGATGAGGAC-3’ |
|  | reverse | 5’-CCAGGCTGAGGTATTCCTTG-3’ |
| Slug | forward | 5’-GGGGAGAAGCCTTTTTCTTG-3’ |
|  | reverse | 5’-TCCTCATGTTTGTGCAGGAG-3’ |
| E-cadherin | forward | 5’-TGCCCAGAAAATGAAAAAGG-3’ |
|  | reverse | 5’-GTGTATGTGGCAATGCGTTC-3’ |
| α-catenin | forward | 5’-AGCGAATTGTGGCAGAGTGT-3’ |
|  | reverse | 5’-GTCTACGCAAGTCCCTGGTC-3’ |
| β-catenin | forward | 5’-ACAACTGTTTTGAAAATCCA-3’ |
|  | reverse | 5’-CGAGTCATTGCATACTGTCC-3’ |
| CD44 | forward | 5’-TTGCAGTCAACAGTCGAAGAAG-3’ |
|  | reverse | 5’-CCTTGTTCACCAAATGCACCA-3’ |
| Oct4 | forward | 5’-CTTGCTGCAGAAGTGGGTGGAGGAA-3’ |
|  | reverse | 5’-CTGCAGTGTGGGTTTCGGGCA-3’ |
| CD133 | forward | 5’-TGGATGCAGAACTTGACAACGT-3’ |
|  | reverse | 5’-ATACCTGCTACGACAGTCGTGGT-3’ |
| CD24 | forward | 5’-TGAAGAACATGTGAGAGGTTTGAC-3’ |
|  | reverse | 5’-GAAAACTGAATCTCCATTCCACAA-3’ |
| CD166 | forward | 5’-TCCTGCCGTCTGCTCTTCT-3’ |
|  | reverse | 5’-TTCTGAGGTACGTCAAGTCGG-3’ |
| SOX2 | forward | 5’-GCCGATGTGAAACTTTTGTCG-3’ |
|  | reverse | 5’-GGCAGCGTGACTTATCCTTCT-3 |
| Nanog | forward | 5’-AATACCTCAGCCTCCAGCAGATG-3’ |
|  | reverse | 5’-TGCGTCACACCATTGCTATTCTTC-3’ |
| GAPDH | forward | 5’-TGCACCACCAACTGCTTAGC-3’ |
|  | reverse | 5’-GGCATGGACTGTGGTCATGAG-3’ |
| U6 | forward | 5’-CTCGCTTCGGCA GCACA-3’ |
|  | reverse | 5’-AACGCTTCACGAATT TGCGT-3’ |
