# Supplementary material for: The circular RNA circDLG1 promotes gastric cancer progression and anti-PD-1 resistance through the regulation of CXCL12 by sponging miR-141-3p
Source: Mol Cancer. 2021 Dec 15;20:166. doi: 10.1186/s12943-021-01475-8 (PMC8672580; doi:10.1186/s12943-021-01475-8)
Supplement: Supplementary file 2 — Additional file 2: Table S2. The information for antibodies used in flow cytometry. [file 12943_2021_1475_MOESM2_ESM.docx]

**Table S2** The information for antibodies used in flow cytometry.

| **Antibodies** | **Company** | **Clone/catalog number** |
| --- | --- | --- |
| anti-mouse IFNGγ | BD Biosciences | XMG1.2 |
| anti-mouse CD45 | BD Biosciences | 30-F11 |
| anti-mouse Gr-1 | TONBO Biosciences | RB6-8C5 |
| anti-mouse Ly6G | TONBO Biosciences | 1A8 |
| anti-mouse Ly6C | BD Biosciences | AL-21 |
| anti-mouse F4/80 | BioLegend | BM8 |
| anti-human CD11b | TONBO Biosciences | M1/70 |
| anti-human CD33 | BD Biosciences | WM53 |
| anti-human HLA-DR | BD Biosciences | G46-6 |
