# Supplementary material for: The circular RNA circDLG1 promotes gastric cancer progression and anti-PD-1 resistance through the regulation of CXCL12 by sponging miR-141-3p
Source: Mol Cancer. 2021 Dec 15;20:166. doi: 10.1186/s12943-021-01475-8 (PMC8672580; doi:10.1186/s12943-021-01475-8)
Supplement: Supplementary file 3 — Additional file 3. Supplementary methods. [file 12943_2021_1475_MOESM3_ESM.docx]

**Supplementary methods**

**RNA in situ hybridization (ISH)**

CircDLG1 expression in gastric cancer tissues was detected by using in situ hybridization (ISH). Briefly, the paraffin-embedded sections were digested with proteinase K after dewaxing and rehydration, fixed with 4% paraformaldehyde, hybridized with the 5’digoxin-labeled circDLG1 probe (Exiqon, Danmark) at 55 ℃ for 12 hours, and then incubated with anti-digoxin monoclonal antibody (Roche, Switzerland) at 4 ℃ for 12 hours. The sections were finally stained with nitro blue tetrazolium/5-bromo-4-chloro-3-indolyphospate. The positive staining of circDLG1 was examined (blue staining). The staining scores were determined based on both the intensity and proportion of circDLG1-positive cells in 10 random fields under a 40× objective. The proportion of stained tumor cells was graded as follows: 0, no positive cells; 1, ＜10%, 2, 10%-50%; 3, ＞50%. The intensity was recorded as follows: 0, no staining; 1, light blue; 2, blue; 3, dark blue. The staining index (SI) was calculated as: SI = staining intensity × proportion of positive staining cells.

**Sphere formation assay**

A sphere-forming assay was performed suing gastric cancer cell lines. Briefly, cell suspensions (1.0 × 10^3^ cells/well) were seeded in 6-well ultralow attachment plates (Corning Inc. Corning, USA) using serum-free DMEM/F12 (Invitrogen) containing 20 ng/mL of basic fibroblast growth factor (Miltenyi Biotec), 2 mM L-glutamine (Mediatech Inc.), and 20 ng/mL of epidermal growth factor (Miltenyi Biotec, Auburn, USA). After culturing for 7 days, the size and number of tumor spheres were evaluated using microscopy.

**RNA sequencing to identify differentially expressed RNA after circDLG1 knockdown**

RNA sequencing was performed according to our previously described method [1]. Briefly, Total RNA was isolated from cells/tissues using Trizol (invitrogen) according to the manufacturer's protocol. RNA purity was assessed using the Qubit®. Each RNA sample had an A260:A280 ratio above 1.8 and A260:A230 ratio above 2.0. RNA integrity was evaluated using the Agilent 2200 TapeStation (Agilent Technologies, USA) and each sample had a RINe above 7.0. Briefly, rRNAs were removed from Total RNA using Epicentre Ribo-Zero rRNARemoval Kit (illumina, USA) and fragmented to approximately 200 bp. Subsequently, the purified RNAs were subjected to first strand and second strand cDNA synthesis followed by adaptor ligation and enrichment with a low-cycle according to instructions of TruSeq® RNA LT/HT Sample Prep Kit (Illumina, USA). The purified library products were evaluated using the Agilent 2200 TapeStation and Qubit®2.0 (Life Technologies, USA) and then diluted to 10 pM for cluster generation in situ on the HiSeq3000 pair-end flow cell followed by sequencing (2×150 bp) on HiSeq3000. Differentially expressed RNAs were evaluated by using the DEGseq algorithm. The differentially expressed circRNAs were defined as q-value < 0.05 with a fold change > 2 or < 0.5.

**Sequencing of tumor tissues and Tumor mutation burden (TMB) analysis**

Sequencing of tumor tissues and Tumor mutation burden (TMB) analysis was performed according to our previously described method [2]. The median level of TMB was used as the cut-off value to divide high and low TMB.

**References:**

1. Chen DL, Lu YX, Zhang JX, Wei XL, Wang F, Zeng ZL , et al. Long non-coding RNA UICLM promotes colorectal cancer liver metastasis by acting as a ceRNA for microRNA-215 to regulate ZEB2 expression. Theranostics 2017; 7(19):4836-4849.

2. Jin Y, Chen DL, Wang F, Yang CP, Chen XX, You JQ , et al. The predicting role of circulating tumor DNA landscape in gastric cancer patients treated with immune checkpoint inhibitors. Mol Cancer 2020; 19(1):154.
