# Supplementary figures and images for: The circular RNA circDLG1 promotes gastric cancer progression and anti-PD-1 resistance through the regulation of CXCL12 by sponging miR-141-3p
Source: Mol Cancer. 2021 Dec 15;20:166. doi: 10.1186/s12943-021-01475-8 (PMC8672580; doi:10.1186/s12943-021-01475-8)

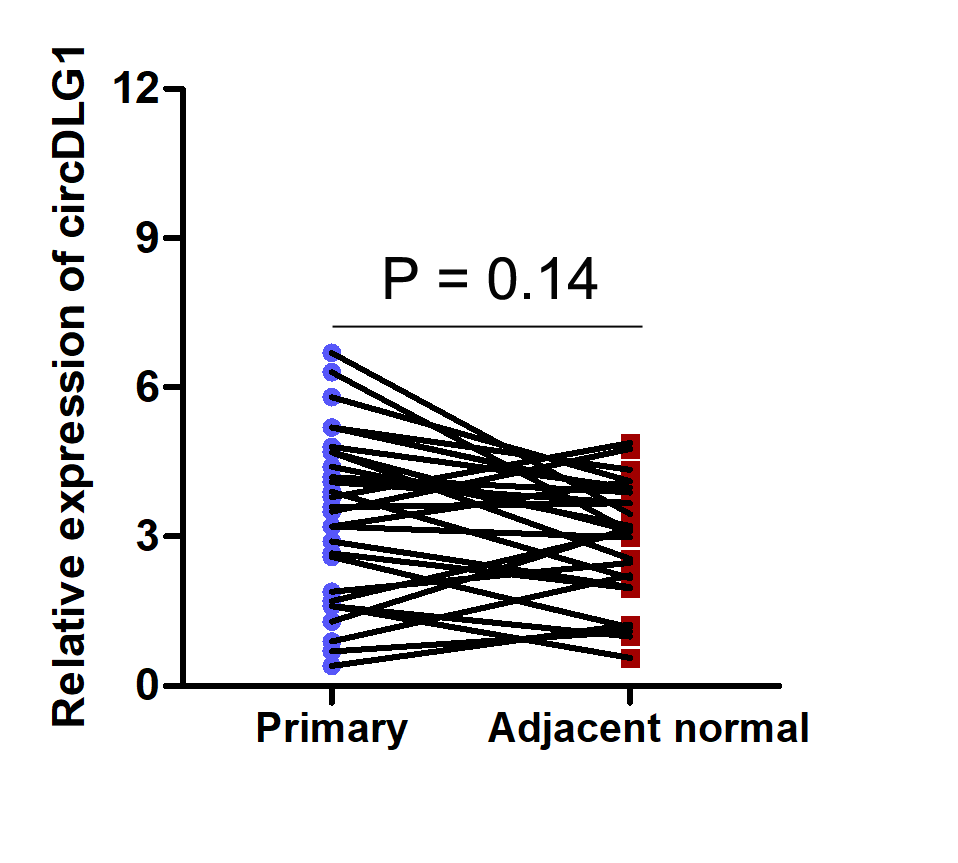

Supplement: Supplementary file 4 — Additional file 4: Figure S1. RT–qPCR analysis of circDLG1 expression in primary gastric cancer tissues and adjacent normal tissues (n = 30, P = 0.14). P = 0.14, Paired Student’s t-test. The data are representative of three technical replicates. [file 12943_2021_1475_MOESM4_ESM.tif]

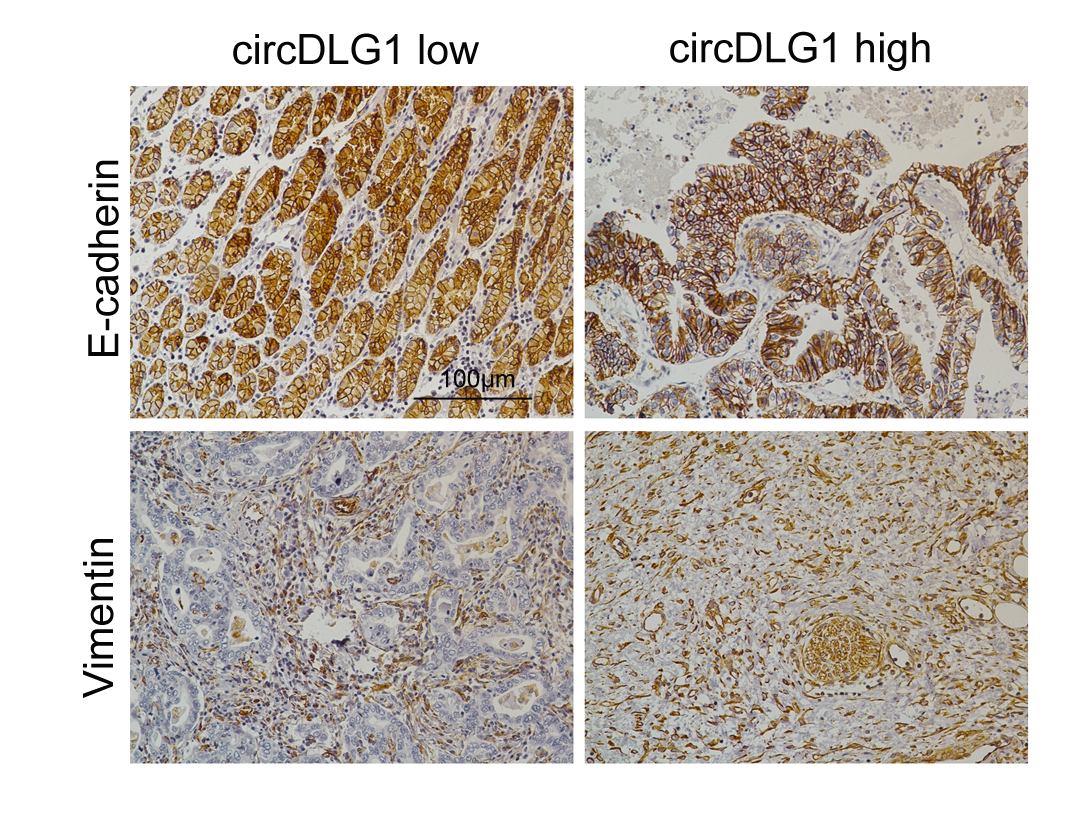

Supplement: Supplementary file 6 — Additional file 6: Figure S2. IHC analysis of E-cadherin and vimentin expression in primary gastric cancer tissues with high and low circDLG1 expression. Scale bar, 100 μm. [file 12943_2021_1475_MOESM6_ESM.tif]

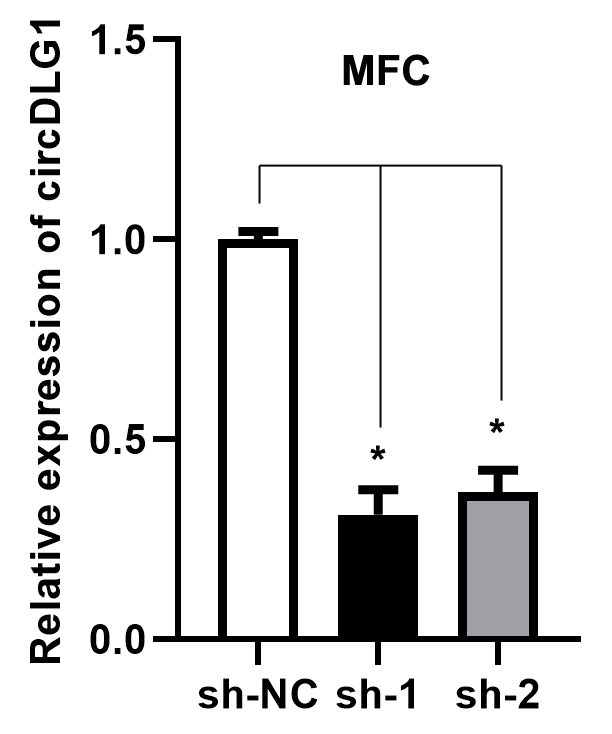

Supplement: Supplementary file 7 — Additional file 7: Figure S3. circDLG1 expression in MFC cells treated with shRNAs. *P < 0.05, one-way ANOVA test for the comparison of the three groups, followed by Student’s t-test for the comparison of sh-1 and sh-2 group with sh-NC group, respectively; multigroup comparisons were adjusted using the Bonferroni method. The experiment was repeated three times. [file 12943_2021_1475_MOESM7_ESM.tif]

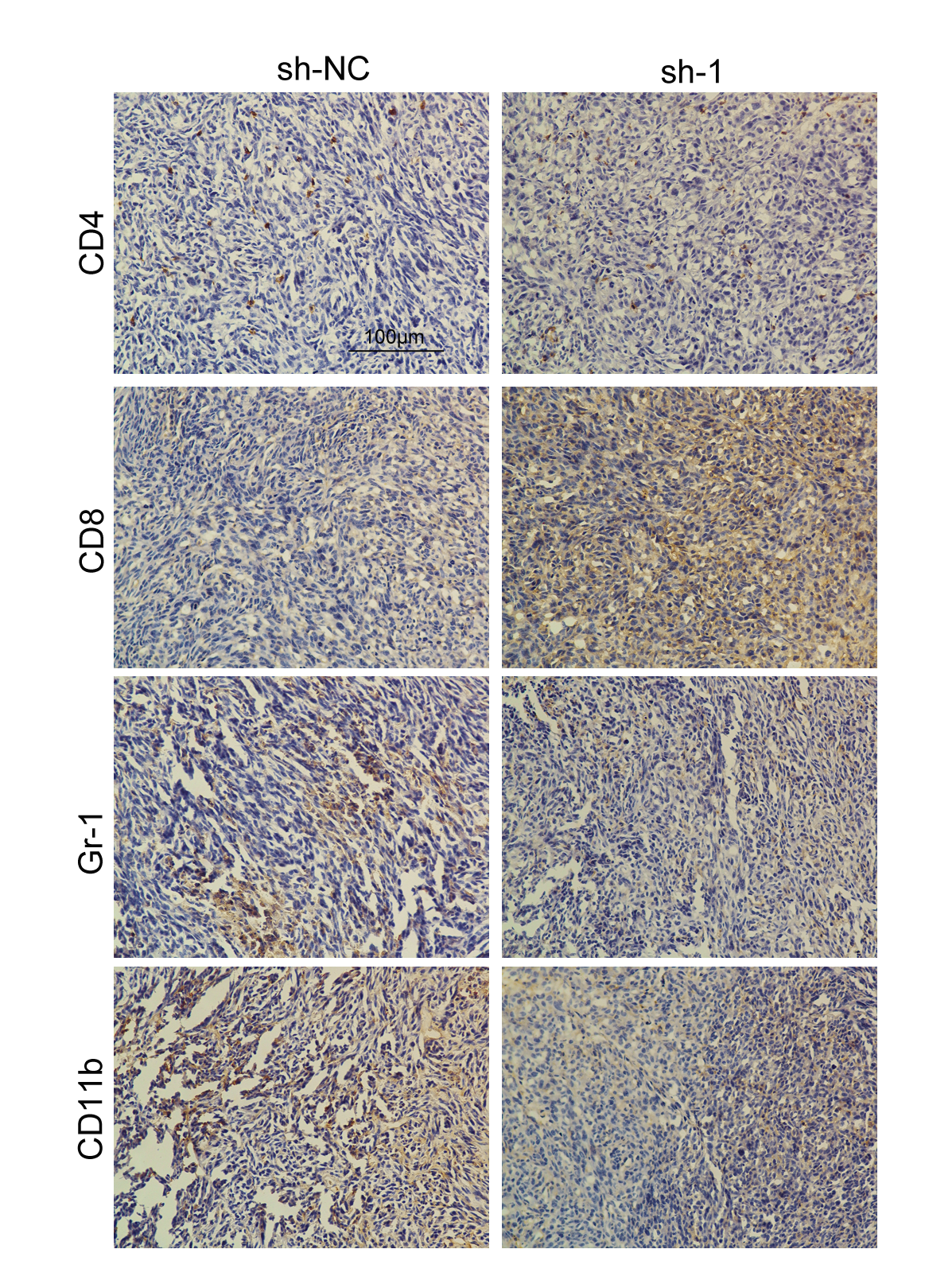

Supplement: Supplementary file 8 — Additional file 8: Figure S4. IHC analysis of CD4, CD8, Gr-1, CD11b expression in MFC-sh-circDLG1-derived tumors and MFC-sh-NC-derived tumors. Scale bar, 100 μm. [file 12943_2021_1475_MOESM8_ESM.tif]

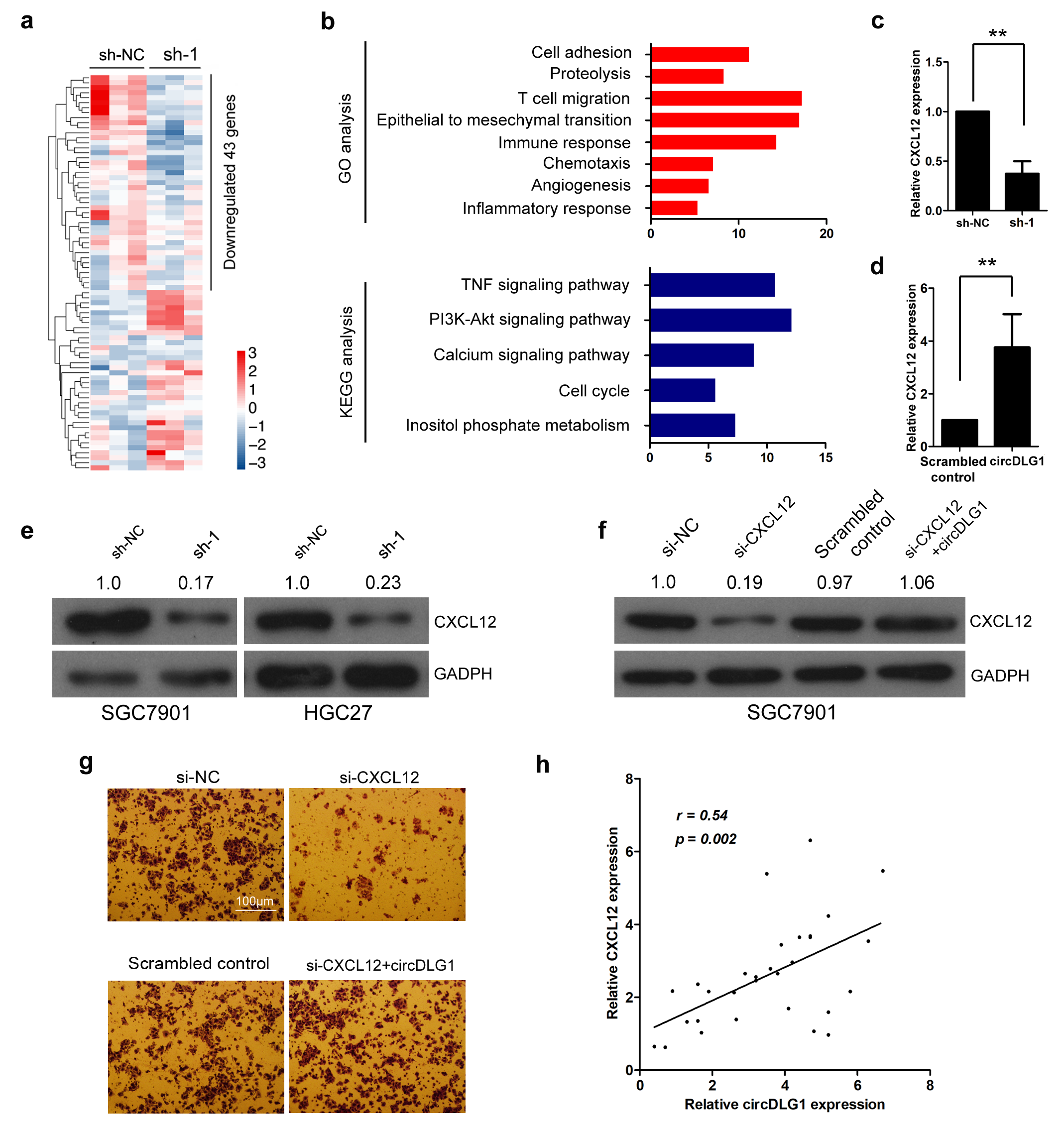

Supplement: Supplementary file 9 — Additional file 9: Figure S5. CircDLG1 upregulates CXCL12 to promote cell progression and immune evasion in gastric cancer. a, A gene expression profile was performed in gastric cancer cells with or without circDLG1 knockdown. SGC7901 cells were transfected with circDLG1 shRNA, and the expression level of mRNAs was detected by RNA sequencing in SGC7901 cells. b, Gene annotation enrichment analysis showed that differentially expressed genes involved in T cell migration, EMT and immune responses were significantly enriched based on the GO analysis, and TNF signaling and the PI3K-Akt signaling pathway were significantly enriched based on the KEGG analysis. c, qRT–PCR analysis of CXCL12 expression in SGC7901 gastric cancer cells after knockdown of circDLG1. **P < 0.01, Student’s t-test; the experiment was repeated three times. d, qRT–PCR analysis of CXCL12 expression in SGC7901 gastric cancer cells after ectopic expression of circDLG1. **P < 0.01, Student’s t-test; the experiment was repeated three times. e, Western blot analysis of CXCL12 in SGC7901 and HGC27 gastric cancer cells after knockdown of circDLG1. f, Western blot analysis of CXCL12 in SGC7901 cells treated with different vectors. g, The cell migration ability of SGC7901 gastric cancer cells treated with different vectors. Scale bar, 100 μm. h, The association between circDLG1 expression and CXCL12 expression as measured by qRT–PCR in gastric cancer tissues. n = 30, P = 0.002, Pearson correlation analysis. [file 12943_2021_1475_MOESM9_ESM.tif]

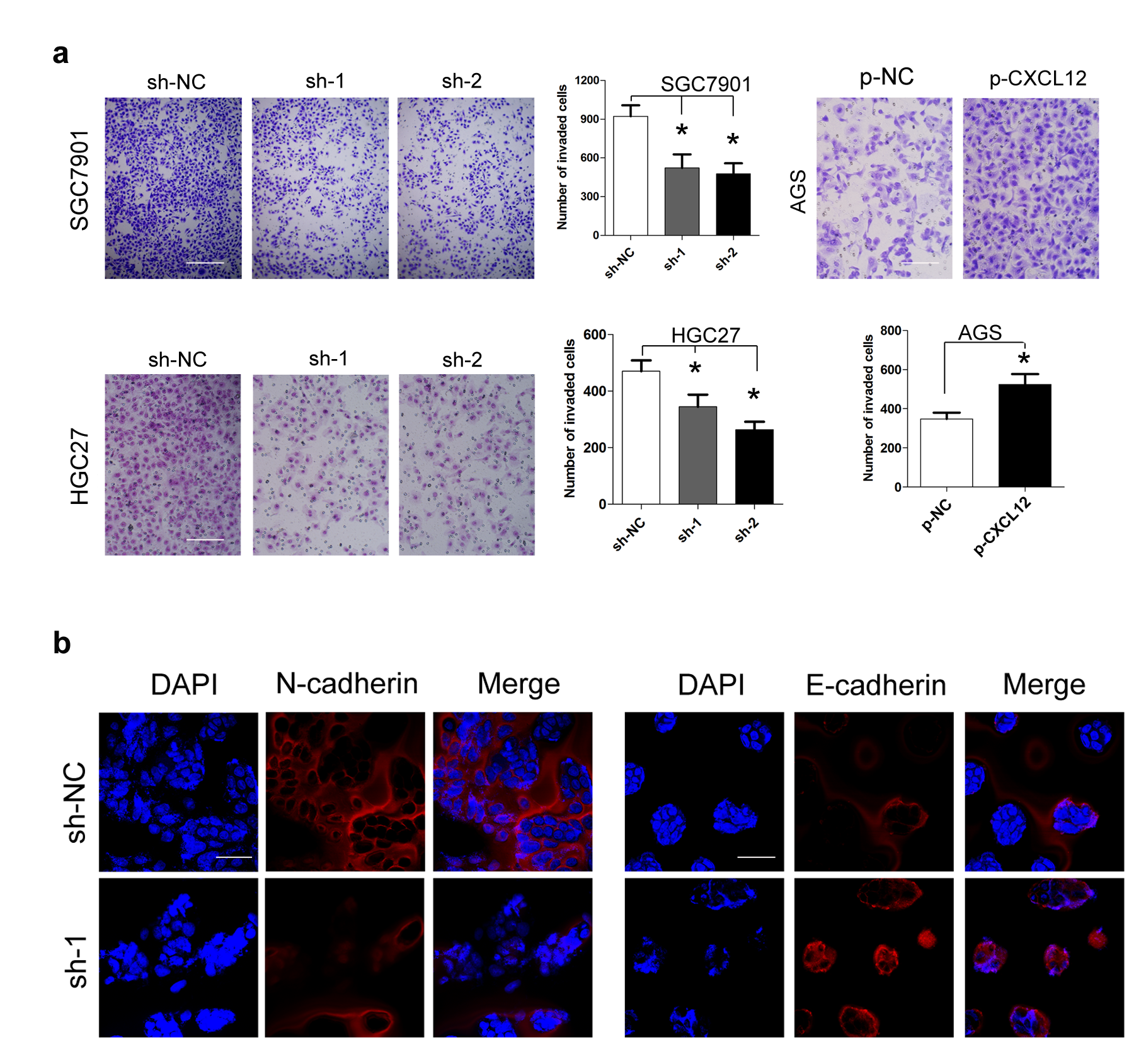

Supplement: Supplementary file 11 — Additional file 11: Figure S6. CXCL12 is associated with an aggressive tumor phenotype in gastric cancer cells. a, The invasion ability of the gastric cancer cell lines SGC7901 and HGC27 after knockdown of CXCL12. *P < 0.05, one-way ANOVA test for the comparison of the three groups, followed by Student’s t-test for the comparison of sh-1 and sh-2 group with sh-NC group, respectively; multigroup comparisons were adjusted using the Bonferroni method. The invasion ability of AGS cells treated with the CXCL12 ectopic expression vector. *P < 0.05, Student’s t-test. Scale bar, 100 μm. b, FISH analysis of N-cadherin and E-cadherin expression after knockdown of CXCL12 in SGC7901 cells. Scale bar, 50 μm. [file 12943_2021_1475_MOESM11_ESM.tif]
