# Supplementary material for: The circular RNA circDLG1 promotes gastric cancer progression and anti-PD-1 resistance through the regulation of CXCL12 by sponging miR-141-3p
Source: Mol Cancer. 2021 Dec 15;20:166. doi: 10.1186/s12943-021-01475-8 (PMC8672580; doi:10.1186/s12943-021-01475-8)
Supplement: Supplementary file 5 — Additional file 5: Table S3. The association between clinicopathologic parameters and circDLG1 expression in 30 gastric cancer patients. [file 12943_2021_1475_MOESM5_ESM.docx]

**Table S3** The association between clinicopathologic parameters and circDLG1 expression in 30 gastric cancer patients.

| Characteristics | n | High expression | Low expression | *P* value |
| --- | --- | --- | --- | --- |
| Age |  |  |  | 0.269 |
| <60 | 13 | 5 | 8 |  |
| ≥60 | 17 | 10 | 7 |  |
| Gender |  |  |  | 0.256 |
| Male | 19 | 11 | 8 |  |
| Female | 11 | 4 | 7 |  |
| Tumor size |  |  |  | 0.025 |
| <4cm | 12 | 3 | 9 |  |
| ≥4cm | 18 | 12 | 6 |  |
| Tumor cell differentiation |  |  |  | 0.107 |
| Well | 2 | 0 | 2 |  |
| Moderate | 7 | 2 | 5 |  |
| Poor | 21 | 13 | 8 |  |
| Peritoneal metastasis |  |  |  | 0.010 |
| Absent | 13 | 3 | 10 |  |
| Present | 17 | 12 | 5 |  |
| Lauren classification |  |  |  | 0.113 |
| Intestinal type | 5 | 1 | 4 |  |
| Diffuse type | 11 | 8 | 3 |  |
| Mixed type | 14 | 6 | 8 |  |

Chi-square test
