# Supplementary material for: The circular RNA circDLG1 promotes gastric cancer progression and anti-PD-1 resistance through the regulation of CXCL12 by sponging miR-141-3p
Source: Mol Cancer. 2021 Dec 15;20:166. doi: 10.1186/s12943-021-01475-8 (PMC8672580; doi:10.1186/s12943-021-01475-8)
Supplement: Supplementary file 10 — Additional file 10: Table S4. The potential miRNAs that could bind to has_circ_0008583 (circDLG1). [file 12943_2021_1475_MOESM10_ESM.docx]

**Table S4** The potential miRNAs that could bind to has_circ_0008583 (circDLG1).

| **circbase ID** | **length** | **miRNA ID (miR_ID)** | **miRanda binding  site (positions)** | **targetscan binding  site (positions)** |
| --- | --- | --- | --- | --- |
| hsa_circ_0008583 | 496 | hsa-miR-3064-5p | 50 | 471 67 477 74 |
| hsa_circ_0008583 | 496 | hsa-miR-3190-5p | 329 | 341 48 347 54 |
| hsa_circ_0008583 | 496 | hsa-miR-4660 | 281 | 298 323 304 329 |
| hsa_circ_0008583 | 496 | hsa-miR-4794 | 455 | 470 68 477 74 |
| hsa_circ_0008583 | 496 | hsa-miR-651-3p | 192 | 208 38 214 43 |
| hsa_circ_0008583 | 496 | hsa-miR-664a-5p | 452 204 | 470 68 477 74 |
| hsa_circ_0008583 | 496 | hsa-miR-1252-5p | 194 | 38 44 |
| hsa_circ_0008583 | 496 | hsa-miR-1301-3p | 304 | 323 329 |
| hsa_circ_0008583 | 496 | hsa-miR-138-5p | 41 | 55 62 |
| hsa_circ_0008583 | 496 | hsa-miR-141-3p | 266 | 284 290 |
| hsa_circ_0008583 | 496 | hsa-miR-153-5p | 320 | 333 339 |
| hsa_circ_0008583 | 496 | hsa-miR-155-3p | 161 | 176 182 |
| hsa_circ_0008583 | 496 | hsa-miR-200a-3p | 273 | 284 290 |
| hsa_circ_0008583 | 496 | hsa-miR-3150a-3p | 59 | 77 83 |
| hsa_circ_0008583 | 496 | hsa-miR-345-3p | 431 | 446 452 |
| hsa_circ_0008583 | 496 | hsa-miR-3652 | 55 | 65 72 |
| hsa_circ_0008583 | 496 | hsa-miR-374a-5p | 255 | 269 275 |
| hsa_circ_0008583 | 496 | hsa-miR-374b-5p | 255 | 269 275 |
| hsa_circ_0008583 | 496 | hsa-miR-376c-3p | 474 | 487 493 |
| hsa_circ_0008583 | 496 | hsa-miR-3922-5p | 144 | 159 165 |
| hsa_circ_0008583 | 496 | hsa-miR-3925-5p | 442 | 457 463 |
| hsa_circ_0008583 | 496 | hsa-miR-452-5p | 267 | 281 287 |
| hsa_circ_0008583 | 496 | hsa-miR-4653-5p | 278 | 294 300 |
| hsa_circ_0008583 | 496 | hsa-miR-4662b | 17 | 33 39 |
| hsa_circ_0008583 | 496 | hsa-miR-4667-5p | 60 | 78 84 |
| hsa_circ_0008583 | 496 | hsa-miR-4738-3p | 66 | 82 88 |
| hsa_circ_0008583 | 496 | hsa-miR-4742-3p | 367 | 382 388 |
| hsa_circ_0008583 | 496 | hsa-miR-487a-5p | 3 | 19 25 |
| hsa_circ_0008583 | 496 | hsa-miR-487b-5p | 5 | 19 25 |
| hsa_circ_0008583 | 496 | hsa-miR-5003-3p | 117 | 130 136 |
| hsa_circ_0008583 | 496 | hsa-miR-504-5p | 432 | 448 455 |
| hsa_circ_0008583 | 496 | hsa-miR-5047 | 309 | 323 329 |
| hsa_circ_0008583 | 496 | hsa-miR-505-3p | 270 | 287 293 |
| hsa_circ_0008583 | 496 | hsa-miR-548ad-3p | 376 | 389 395 |
| hsa_circ_0008583 | 496 | hsa-miR-5583-5p | 429 | 441 447 |
| hsa_circ_0008583 | 496 | hsa-miR-637 | 62 | 79 84 |
| hsa_circ_0008583 | 496 | hsa-miR-638 | 463 | 480 486 |
| hsa_circ_0008583 | 496 | hsa-miR-6516-3p | 391 | 406 412 |
| hsa_circ_0008583 | 496 | hsa-miR-653-5p | 155 | 168 174 |
| hsa_circ_0008583 | 496 | hsa-miR-6719-3p | 474 | 490 496 |
| hsa_circ_0008583 | 496 | hsa-miR-6763-5p | 66 | 77 83 |
| hsa_circ_0008583 | 496 | hsa-miR-6807-5p | 138 | 149 156 |
| hsa_circ_0008583 | 496 | hsa-miR-6882-3p | 302 | 316 323 |
| hsa_circ_0008583 | 496 | hsa-miR-7155-3p | 34 | 45 52 |
| hsa_circ_0008583 | 496 | hsa-miR-758-3p | 335 | 351 357 |
| hsa_circ_0008583 | 496 | hsa-miR-7849-3p | 340 | 355 361 |
